# Supplementary material for: Crossover Inhibition Generates Sustained Visual Responses in the Inner Retina
Source: Neuron. 2016 Apr 20;90(2):308–19. doi: 10.1016/j.neuron.2016.03.015 (PMC4848346; doi:10.1016/j.neuron.2016.03.015)
Supplement: Document S1. Figures S1–S5 [file mmc1.pdf]

Neuron, Volume *90*

Supplemental Information

Crossover Inhibition Generates Sustained Visual Responses in the Inner Retina

Juliana M. Rosa, Sabine Ruehle, Huayu Ding, and Leon Lagnado

Crossover-inhibition generates sustained visual responses in the inner retina

Supplementary Figure Legends

Figure S1: Two further functional classes of bipolar cell terminal. Related to Figures 1 and 2.

(A and B). Although the majority of bipolar cell terminals fell into one of the five functional classes shown in Figs. 1 and 2, we also observed two further small but distinct populations: OFF terminals that were suppressed-by-contrast (4%, Fig. S1A; 37 terminals from 7 fish) and others that could not be classified as ON or OFF but displayed band-pass characteristics (Fig. S1B; 13 terminals from 7 fish). *Top*: Raster plot showing the relative changes in fluorescence (ΔF/F). *Bottom*: Averaged SyGCaMP2 response from the same contrast-suppressed and frequency-activated OFF terminals shown in raster plots. SEM indicated in gray. (C) Plot of response amplitude as a function of frequency averaged from the same population of contrast-suppressed OFF terminals (black) and unclassified band-pass terminals (blue; fc = 15.1 ± 1.5 Hz).

Figure S2: The three temporal channels through the OFF pathway are not an artifact generated by activity-dependent adaptation. Related to Figure 1 and 2.

Application of a “reversed” (i.e. from highest to lowest frequency) stimulation generates the same temporal tuning response in OFF terminals observed in Figure 1 and 2.

(A and B) Averaged responses within each of the 3 groups to stimuli of frequency indicated. 94 OFF terminals were sorted into 3 distinct groups according to the K-means clustering shown in B. (C) Plot of response amplitude as a function of frequency for the three groups of OFF terminals shown in A and B. (D) Spatial distribution of each OFF group as a function of layer. OFF terminals in Group 1 (low-pass) were at highest density in layer 6, whereas terminals in Group 3 (band-pass) were predominantly localized in layer 1. OFF bipolar terminals in Group 2 stratified throughout IPL.

Figure S3: The two temporal channels through the ON pathway are not an artifact generated by activity-dependent adaptation. Related to Figure 1 and 2.

Application of a "reversed" (i.e. from highest to lowest frequency) stimulation generates the same temporal tuning response in ON terminals observed in Figure 1 and 2.

(A) Averaged responses from 60 ON contrast-activated (green) and 70 ON contrast-suppressed (black) terminals. Light stimulation is shown in grey. (B) Plot of response amplitude as a function of frequency for the two groups of ON terminals shown in A. (C) Spatial distribution of contrast-activated and contrast-suppressed ON bipolar terminals as a function of layer. The depth of the terminal in the IPL was measured from the photoreceptor side (layer 1) to ganglion cells (layer 6).

Figure S4: Effect of crossover inhibition according to layers of the IPL. Related to Figures 3 and 4.

(A) As shown in Fig. 3, layer 6 contained the highest density of sustained OFF terminals in Group 1 (low-pass) under control conditions: blocking ON signals with L-AP4 almost completely abolished these sustained responses while simultaneously increasing the

density of terminals in Group 2. This effect also manifested itself as an almost complete abolition of the response to a step of light (B) and a shift in the tuning curve averaged over all OFF terminals from low-pass to band-pass (C). Notably, none of these three effects was apparent in the responses of OFF terminals in Layer 1 (D), which is almost completely devoid of an ON input. These results reveal a previously unrecognized role of crossover inhibition: the conversion of OFF synapses with band-pass characteristics into low-pass filters.

Figure S5: Effects of glycinergic and GABAergic inhibition on the gain of transmission through the three major OFF temporal channels. Related to Figure 5.

(A) Plot of response amplitude as a function of frequency averaged over the same populations of OFF terminals shown in Figure 5A-B, before (red) and after gabazine (black) or strychnine (blue). No significant changes were detected in the amplitude and in the tuning of all three groups.

Figure S1

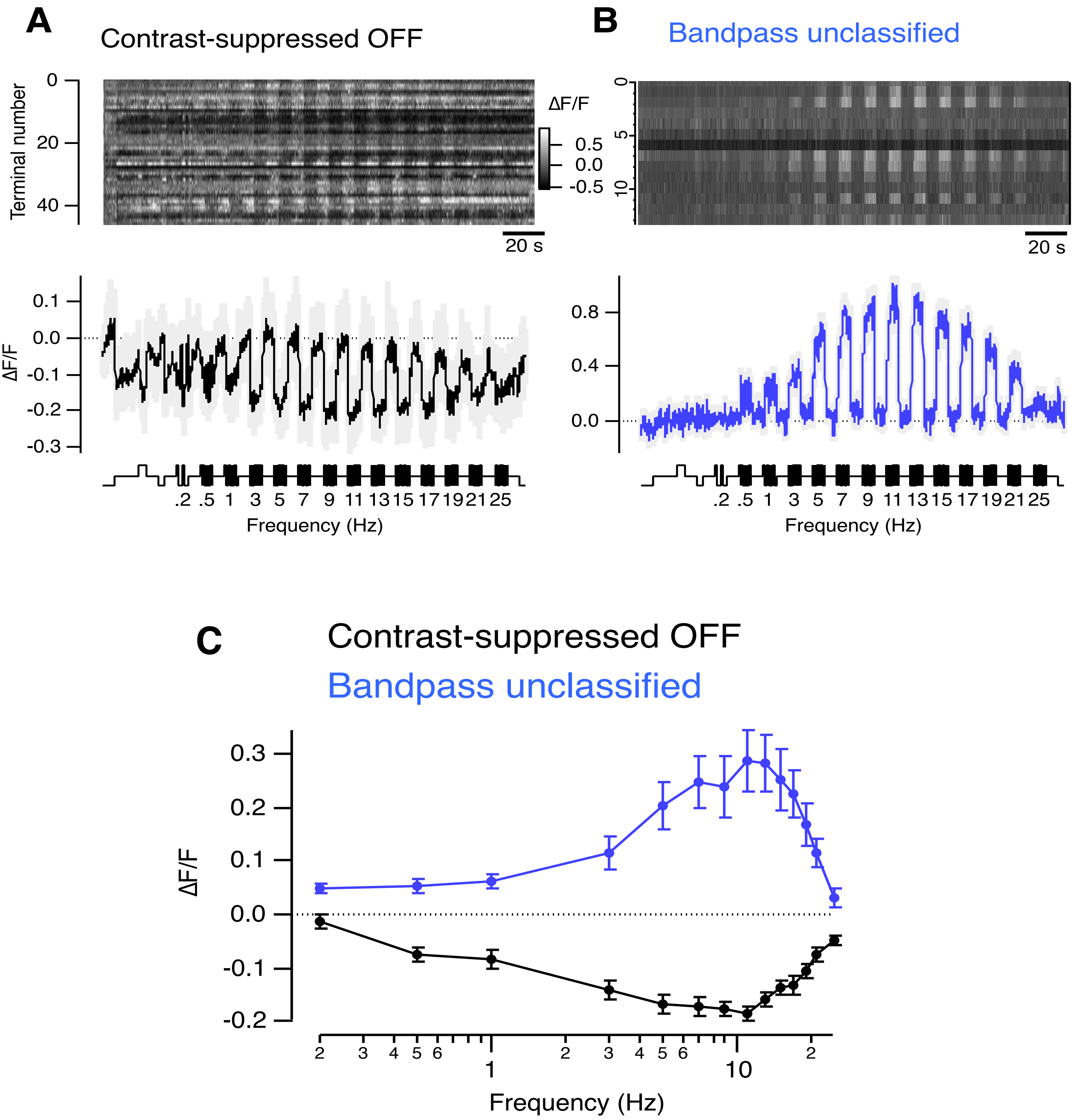
A
Contrast-suppressed OFF
Terminal number
0
20
40
ΔF/F
0.5
0.0
-0.5
20 s
0.1
0.0
-0.1
-0.2
-0.3
.2 .5 1 3 5 7 9 11 13 15 17 19 21 25
Frequency (Hz)
B
Bandpass unclassified
0
5
10
20 s
0.8
0.4
0.0
.2 .5 1 3 5 7 9 11 13 15 17 19 21 25
Frequency (Hz)
C
Contrast-suppressed OFF
Bandpass unclassified
0.3
0.2
0.1
0.0
-0.1
-0.2
2 3 4 5 6 1 2 3 4 5 6 10 2
Frequency (Hz)

Figure S2

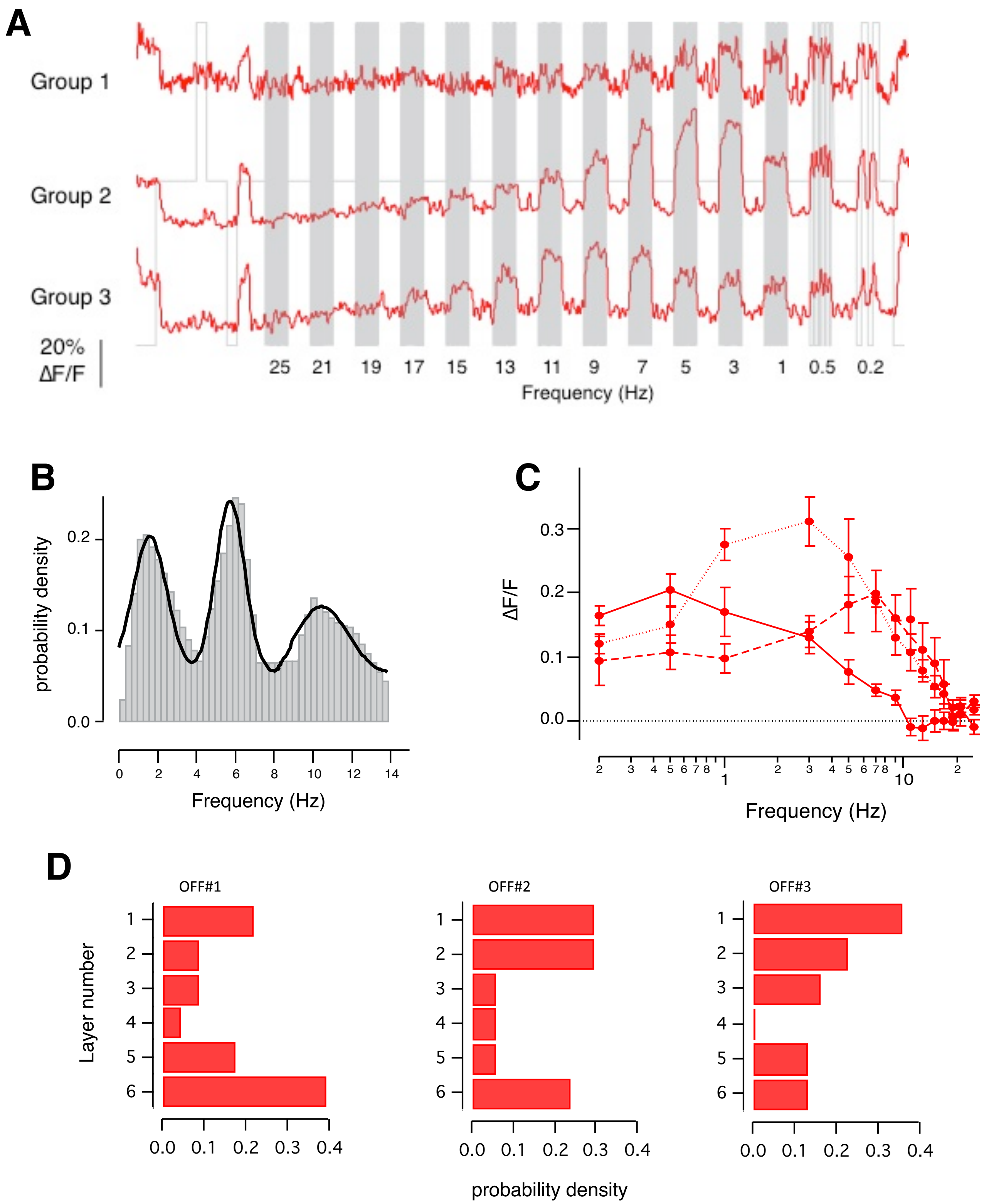

A
Group 1
Group 2
Group 3
20% ΔF/F
25 21 19 17 15 13 11 9 7 5 3 1 0.5 0.2
Frequency (Hz)
B
probability density
0.0 0.1 0.2
0 2 4 6 8 10 12 14
Frequency (Hz)
C
ΔF/F
0.0 0.1 0.2 0.3
2 3 4 5 6 7 8 1 2 3 4 5 6 7 8 10 2
Frequency (Hz)
D
OFF#1
OFF#2
OFF#3
Layer number
1 2 3 4 5 6
0.0 0.1 0.2 0.3 0.4
probability density

Figure S3

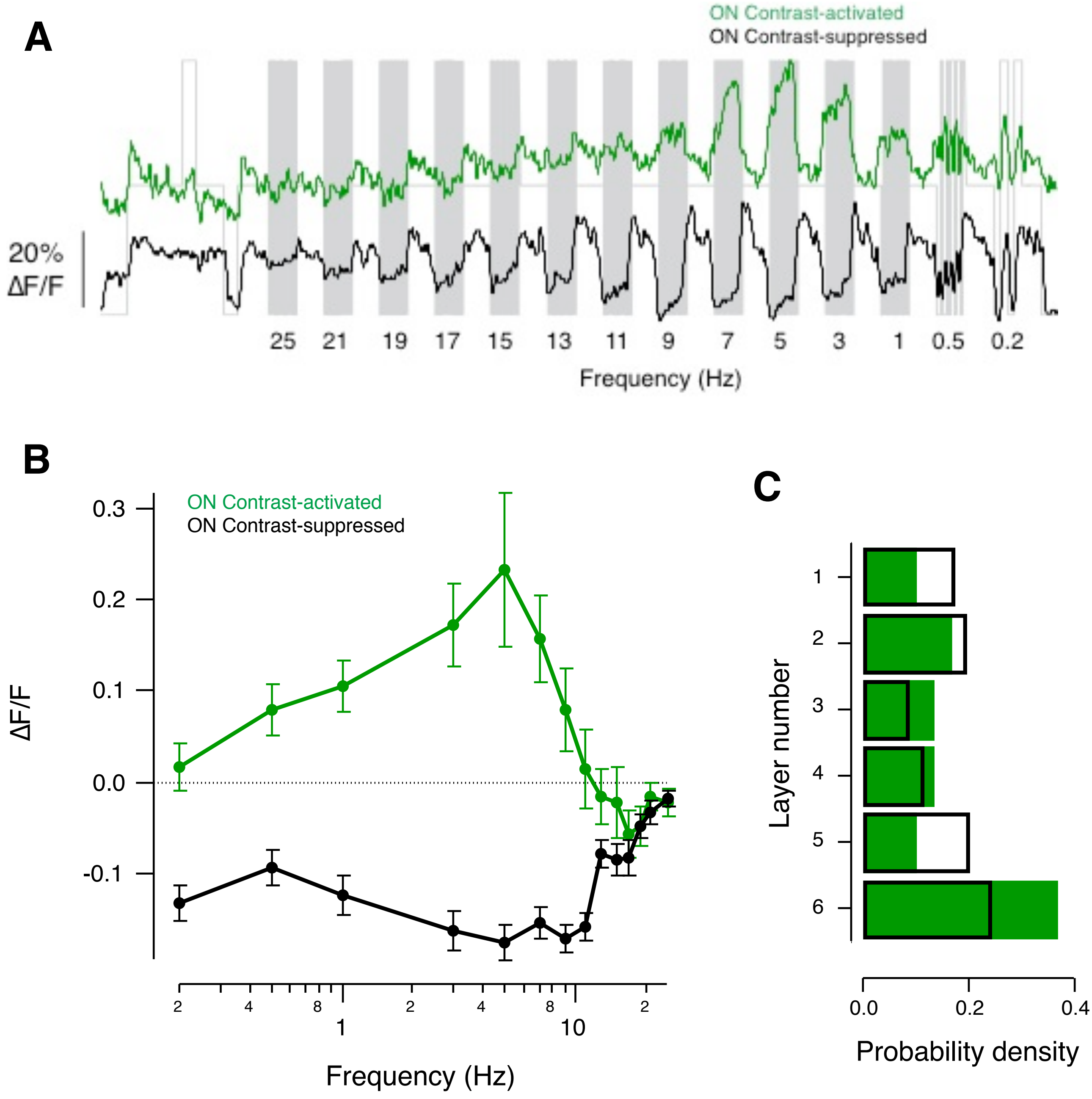
A
ON Contrast-activated
ON Contrast-suppressed
20% ΔF/F
25 21 19 17 15 13 11 9 7 5 3 1 0.5 0.2
Frequency (Hz)
B
ON Contrast-activated
ON Contrast-suppressed
ΔF/F
0.3
0.2
0.1
0.0
-0.1
2 4 8 1 2 4 8 10 2
Frequency (Hz)
C
Layer number
1
2
3
4
5
6
0.0 0.2 0.4
Probability density

Figure S4

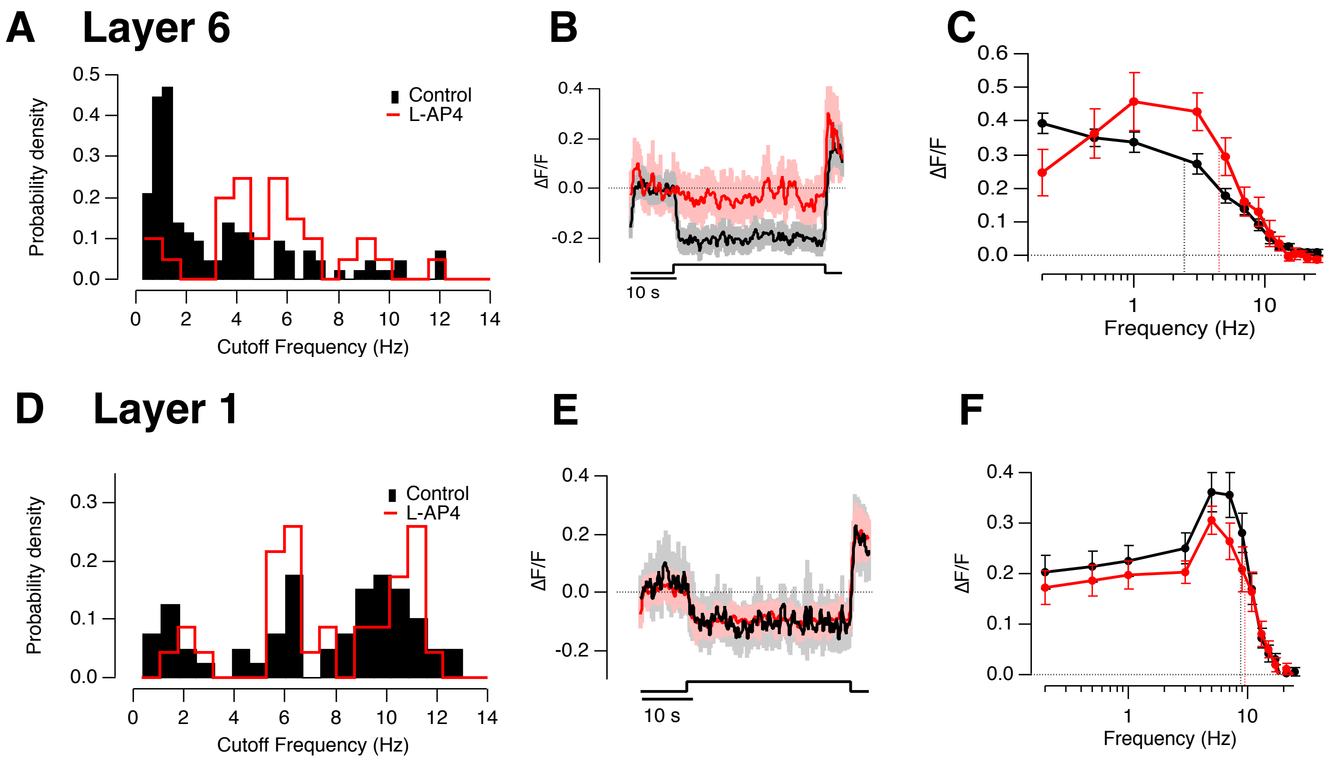
A
Layer 6
Probability density
Control
L-AP4
Cutoff Frequency (Hz)
B
ΔF/F
10 s
C
ΔF/F
Frequency (Hz)
D
Layer 1
Probability density
Control
L-AP4
Cutoff Frequency (Hz)
E
ΔF/F
10 s
F
ΔF/F
Frequency (Hz)

Figure S5

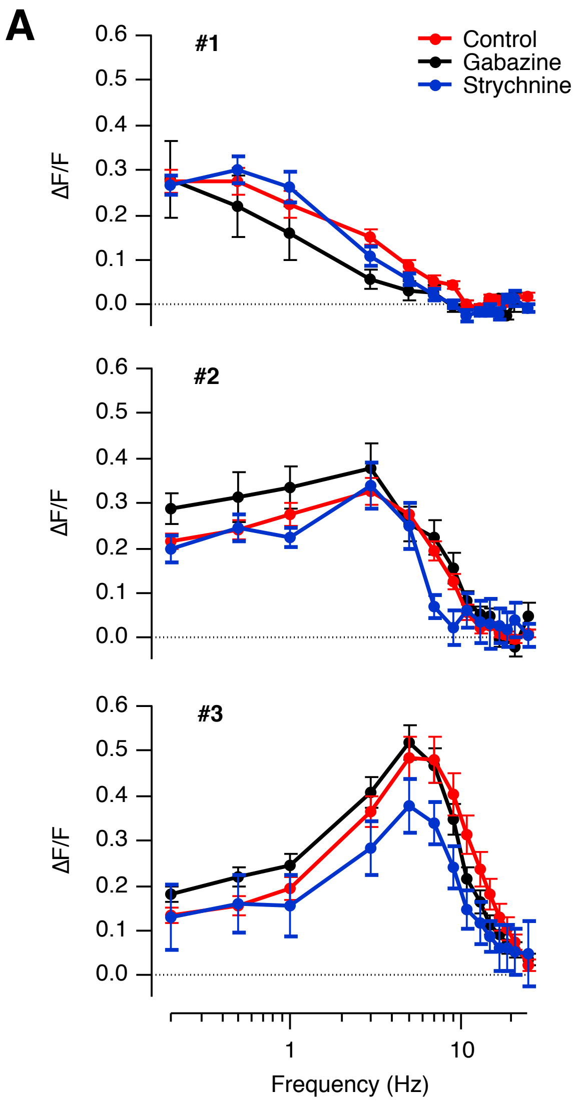
A
#1
#2
#3
Control
Gabazine
Strychnine
ΔF/F
0.6
0.5
0.4
0.3
0.2
0.1
0.0
1
10
Frequency (Hz)
