# Supplementary material for: Crossover Inhibition Generates Sustained Visual Responses in the Inner Retina
Source: Neuron. 2016 Apr 20;90(2):308–19. doi: 10.1016/j.neuron.2016.03.015 (PMC4848346; doi:10.1016/j.neuron.2016.03.015)
Supplement: Document S2. Article plus Supplemental Information [file mmc2.pdf]

Article

Neuron

Crossover Inhibition Generates Sustained Visual Responses in the Inner Retina

Highlights

- Bipolar cells transmit the visual signal through at least five temporal channels
- Crossover inhibition from the ON pathway generates the sustained OFF channel
- GABAergic inhibition converts band-pass synapses into low-pass
- Glycinergic inputs to ON terminals generate "uniformity detectors"

Authors

Juliana M. Rosa, Sabine Ruehle, Huayu Ding, Leon Lagnado

Correspondence

l.lagnado@sussex.ac.uk

In Brief

By imaging synaptic activity in vivo, Rosa et al. determined the basic circuitry by which the sustained channel is generated in the inner retina. The authors show that amacrine cells tune the bipolar cell output to lower frequencies and define a new function of crossover inhibition: the generation of sustained OFF signals.

CrossMark

Rosa et al., 2016, Neuron *90*, 308–319
April 20, 2016 ©2016 The Authors
http://dx.doi.org/10.1016/j.neuron.2016.03.015

CellPress

Neuron
Article

Crossover Inhibition Generates Sustained Visual Responses in the Inner Retina

Juliana M. Rosa,[1,3] Sabine Ruehle,[1,3] Huayu Ding,[1] and Leon Lagnado[1,2,*]
[1]MRC Laboratory of Molecular Biology, Francis Crick Avenue, Cambridge, CB2 0QH, UK
[2]School of Life Sciences, University of Sussex, Brighton, BN1 9QG, UK
[3]Co-first author
*Correspondence: l.lagnado@sussex.ac.uk
http://dx.doi.org/10.1016/j.neuron.2016.03.015

SUMMARY

In daylight, the input to the retinal circuit is provided primarily by cone photoreceptors acting as band-pass filters, but the retinal output also contains neuronal populations transmitting sustained signals. Using in vivo imaging of genetically encoded calcium reporters, we investigated the circuits that generate these sustained channels within the inner retina of zebrafish. In OFF bipolar cells, sustained transmission was found to depend on crossover inhibition from the ON pathway through GABAergic amacrine cells. In ON bipolar cells, the amplitude of low-frequency signals was regulated by glycinergic amacrine cells, while GABAergic inhibition regulated the gain of band-pass signals. We also provide the first functional description of a subset of sustained ON bipolar cells in which synaptic activity was suppressed by fluctuations at frequencies above ~0.2 Hz. These results map out the basic circuitry by which the inner retina generates sustained visual signals and describes a new function of crossover inhibition.

INTRODUCTION

The retina transforms the visual input through a number of parallel channels containing distinct spatio-temporal filters (Masland, 2001, 2012; Roska and Werblin, 2001; Wässle, 2004). Most of these channels are generated by the circuitry of the inner plexiform layer (IPL), which contains the dendrites of about ~30 functional types of retinal ganglion cells (RGCs) stratifying in 5–6 different strata (Roska and Werblin, 2001). In each stratum, RGC dendrites receive excitatory synaptic inputs from bipolar cells with different filtering properties, and at least two distinct temporal filters have been recognized for decades: in the "transient" channel, RGCs receive excitatory inputs from bipolar cells acting as band-pass filters, while in the "sustained" channel, RGCs receive synaptic inputs with low-pass characteristics (Awatramani and Slaughter, 2000). However, only one temporal filter operates on the input to the retinal circuit under normal daylight: the band-pass filter provided by cone photoreceptors (Schnapf et al., 1990). Filtering then remains band-pass as the visual signal is transmitted through the cell body of post-synaptic bipolar cells (Baden et al., 2011), indicating that sustained visual channels are not established until the IPL. Here we ask a fundamental question: how are sustained channels generated by the circuitry of the inner retina?

Superimposed on the separation of temporal channels is a second key aspect of retinal processing—the decomposition of the visual input into two streams of opposing polarity, the ON and OFF pathways, which also originate in bipolar cells. In most species, the output from OFF bipolar cells projects to sublamina *a* of the IPL, while the output from ON signals projects to sublamina *b*. It has long been known that the ON and OFF pathways can merge again onto mixed ON-OFF ganglion cells (Werblin, 2011), but it is now clear that they can also interact within the IPL through inhibitory amacrine cells sending processes through both ON and OFF sublaminae—a process called crossover inhibition (Molnar and Werblin, 2007; Manookin et al., 2008; Baccus, 2007). The roles of crossover inhibition between ON and OFF pathways are still being investigated, but two of the better defined are to allow ganglion cells to continuously signal changes in temporal contrast in the face of changes in the mean luminance (Manookin et al., 2008) and compensating the distorting effects of synaptic rectification (Molnar et al., 2009).

In this study, we investigated how interactions between ON and OFF pathways contribute to the generation of different temporal channels by imaging transmission of the visual signal within the IPL using genetically encoded calcium reporters (Nikolaev et al., 2013; Odermatt et al., 2012; Dreosti et al., 2011; Dorostkar et al., 2010). We find that the OFF pathway transmits sustained signals through a population of bipolar cell synapses that become tuned to lower frequencies by crossover inhibition from ON bipolar cells, with the link made exclusively through GABAergic amacrine cells. The sensitivity of the ON pathway to low-frequency signals also depends on inhibition, but primarily through glycinergic amacrine cells. Additionally, we demonstrate for the first time a subset of ON bipolar cells that act as "uniformity detectors" (Sivyer et al., 2010); the activity of their synaptic outputs are strongly inhibited by any fluctuations at frequencies above ~0.2 Hz, again through the action of glycinergic amacrine cells. These results indicate that there are at least three distinct pathways by which amacrine cells tune the synaptic output of bipolar cells to lower frequencies and define a new function of crossover inhibition—the generation of sustained OFF signals in the inner retina.

This is an open access article under the CC BY license (http://creativecommons.org/licenses/by/4.0/).

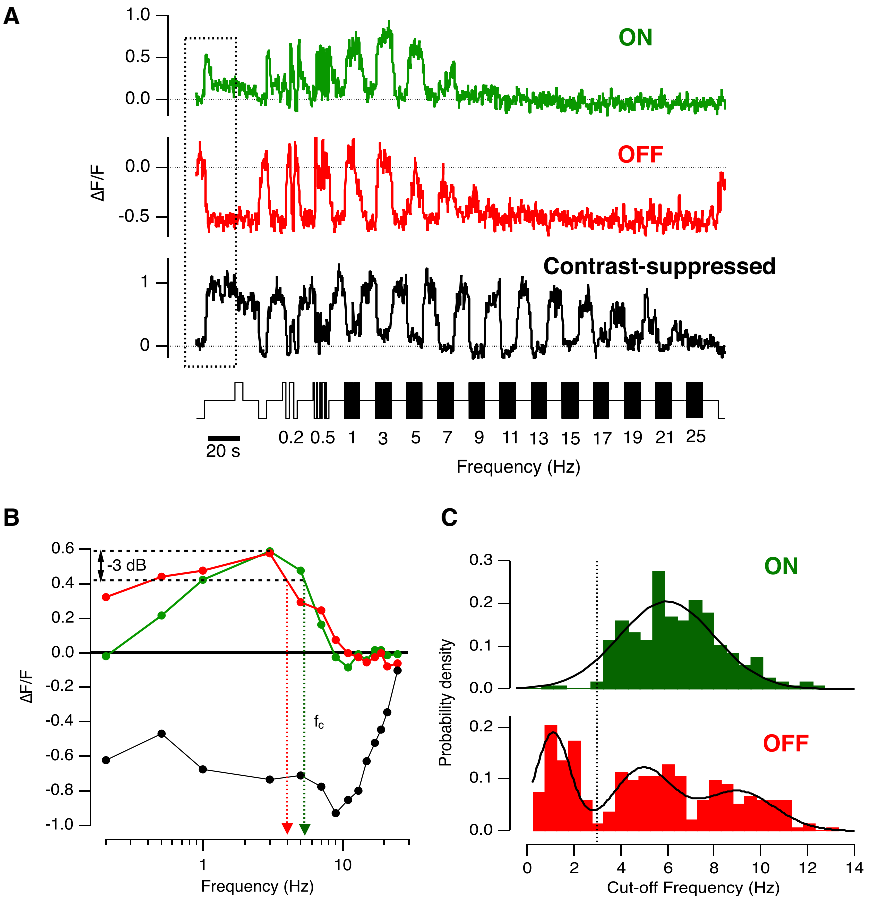

Figure 1. Assessing the Frequency Tuning of Signals Transmitted to the Inner Retina

(A) Examples of SyGCaMP2 responses in three individual bipolar cell terminals. The stimulus (lower trace) consisted of a light step followed by modulation at different frequencies between 0.2 and 25 Hz (90% contrast, square wave, mean intensity 55 nW/mm^2). An ON terminal activated by contrast is shown in green and an OFF terminal in red. An example of an ON terminal inhibited by contrast is shown in black (contrast-suppressed response). The polarity of the terminal was determined in response to an initial step of light shown in the boxed area.

(B) Transfer functions of the individual terminals shown in (A). The response at each frequency was calculated as the average value of change in fluorescence (ΔF/F) during the stimulus. Dotted lines represent cutoff frequencies (fc) at −3 dB of the maximum response for the ON (green) and OFF (red) terminals with values of ~4 Hz and ~5.3 Hz, respectively. Note the band-pass characteristic with attenuation of the response at both low and high frequency in both ON and OFF response. The black trace is the transfer function of the contrast-suppressed terminal shown in (A).

(C) Histogram of the cutoff frequency (fc) of 264 activated-by-contrast ON bipolar terminals (green) and 263 activated-by-contrast OFF bipolar terminals (red) from 7 fish. The function fitted to the distribution of fc in ON terminals is a Gaussian with m = 6.4 Hz and width = 2.7 Hz. The function fitted to the distribution of fc in OFF terminals is the sum of three Gaussians with (m = 1.1 Hz and width = 1 Hz for peak 1), (m = 4.9 Hz and width = 1.7 Hz for peak 2), and (m = 9 Hz and width = 2.2 Hz for peak 3).

RESULTS

Five Frequency-Dependent Channels Transmitting to the Inner Retina

To investigate how visual signals of different frequencies are transferred to the inner retina, we used transgenic zebrafish expressing SyGCaMP2 at ribbon synapses (Dreosti et al., 2009). This approach allows one to image presynaptic calcium transients through the entire population of bipolar cell terminals in the IPL (Dreosti and Lagnado, 2011). A full-field stimulus modulated at frequencies between 0.2 Hz and 25 Hz (90% contrast) elicited strongly rectifying responses from individual bipolar terminals, as described previously (Nikolaev et al., 2013; Esposti et al., 2013). Individual examples of ON and OFF terminals stimulated by an increase in contrast are shown in Figure 1A, together with a third very distinctive class of response—ON terminals *inhibited* by an increase in contrast (which we will refer to as contrast-suppressed terminals). For each of these three examples, we plotted tuning curves, or the amplitude of the synaptic responses as a function of stimulus frequency, in Figure 1B. From these plots, we calculated the cutoff frequency (fc) as −3 dB of the peak amplitude and the histograms in Figure 1C show the distributions of fc for ON and OFF terminals activated by an increase in contrast (n = 264 and 263 terminals, respectively).

The survey in Figure 1 highlighted three fundamental features in the temporal filters operating at the source of the ON and OFF pathways under photopic conditions. First, there were three populations of OFF terminals, with fc values centered on 1.1, 4.9, and 9.0 Hz. Second, ON terminals activated by contrast formed one broad population centered on fc = 6.4 Hz. Third, there were almost no ON terminals with fc below ~3 Hz (dotted line, Figure 1C). These results demonstrate that there are at least five distinct frequency channels through which the visual signal is transmitted to the inner retina: three OFF channels, all activated by temporal contrast, and two ON channels, one activated and one inhibited.

The frequency dependence of responses averaged across the complete population of OFF and ON terminals through all layers of the IPL is shown in further detail in Figure 2. The distribution of cutoff frequencies of the OFF terminals displayed three peaks (Figure 1C), and the K-means algorithm allowed us to recognize the same three groups by clustering the tuning curves measured for each terminal (Figures 2A and 2B). Group 1 terminals (33%) were low-pass, with an average cutoff frequency of 1.9 ± 0.08 Hz; Group 2 (37%) displayed band-pass characteristics, with an average cutoff frequency of 5.5 ± 0.17 Hz; and Group 3 (30%) were tuned more narrowly with an average cutoff at 10 ± 0.22 Hz. The tuning curves measured when gradually increasing

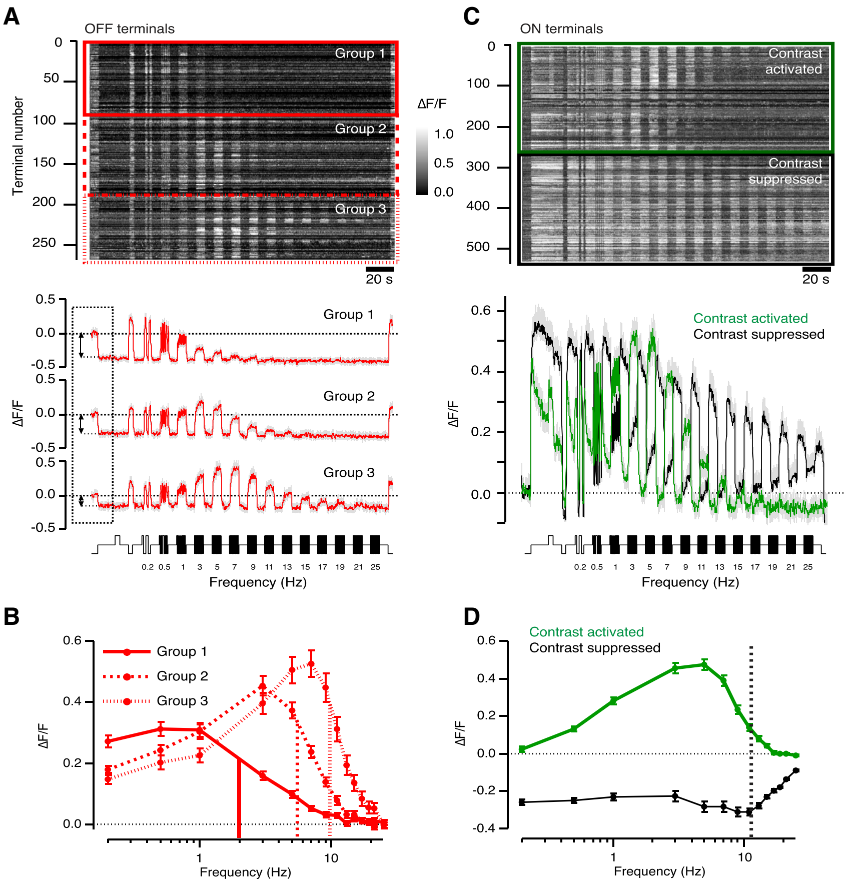

Figure 2. Distinct Temporal Channels through the ON and OFF Pathways

(A) Top: raster plot showing the relative change in fluorescence (ΔF/F) for 264 OFF terminals sorted into 3 distinct groups according to the K-means clustering. Red boxes represent the separation of each group in the raster plot. Bottom: averaged responses within each of the 3 groups to stimuli of frequency indicated. Double arrows indicate the sustained response at light onset, which was largest in Group 1. SEM indicated in gray.

(B) Plot of response amplitude as a function of frequency averaged for each of the three groups of OFF terminals shown in (A). Group 1 terminals behaved as low-pass filters with fc = 1.9 ± 0.08 Hz (n = 88). Groups 2 and 3 behaved as band-pass filters with fc = 5.5 ± 0.17 Hz (n = 97) and fc = 10.1 ± 0.22 Hz (n = 79), respectively.

(C) Top: raster plot showing the relative change in fluorescence (ΔF/F) for contrast-activated (green box; n = 263) and contrast-suppressed ON terminals (black box; n = 277). Bottom: averaged responses from the same populations of contrast-activated (green) and contrast-suppressed (black) terminals. SEM indicated in gray.

(D) Plot of response amplitude as a function of frequency for the two groups of ON terminals shown in (C). The black, dotted line indicates the decrease in the suppression of contrast-suppressed ON terminals.

See also Figures S1–S3.

stimulus frequency were indistinguishable from those measured by gradually decreasing frequency (Figure S2). The various temporal channels that we identified were not, therefore, an artifact generated by activity-dependent adaptation as we varied stimulus frequency.

Of the terminals that could be defined as ON or OFF from the response to a step increment or decrement of light, 95% fell into one of the five functional groups described in Figures 1 and 2. However, two other small but distinct functional classes of bipolar cell terminal were also recognized. First, 4% of terminals within the OFF channel were suppressed rather than activated by contrast (Figures S1A and S1C). Second, about 4% of all terminals did not respond to a step of light and could not be classified as ON or OFF but were nonetheless activated by fluctuations at frequencies higher than ~0.5 Hz (Figures S1B and S1C). The band-pass characteristics of this last group most closely resembled ON terminals activated by contrast shown in Figure 1B. These smaller populations of terminals were not analyzed further in this study.

Here we have characterized transmission of the visual signal by measuring synaptic activation across a range of frequencies. There is a direct relation between this approach and the simpler characterization of the "transient" and "sustained" channels according to the decay kinetics of the response to a step of light. Comparing the step responses at the start of the traces in Figures 1 and 2 to the complete tuning curves, it can be seen that ON terminals show the fastest and largest decay (Figure 1A), as well as the most strongly band-pass tuning curves (Figures 1B and 2D). OFF terminals did not display a clearly decaying response to a light decrement (Figure 2A), but the amplitude of the step response was larger in Group 1 (low-pass) compared to Groups 2 and 3 (band-pass), which is in line with the amplitude of the low-frequency asymptotes of the tuning curves in Figure 2B. The lack of a clear decay in the step response of OFF terminals may reflect the rectifying relationship between membrane potential and intracellular calcium. A recent study using calcium imaging in the retina of mice was able to detect a decaying response to a light step in some OFF bipolar cell terminals, but the clearest distinction between these "transient" neurons compared to the "sustained" terminals was again found in the amplitude of the step response rather than its kinetics (Baden et al., 2014).

Contrast-Suppressed Responses through the ON Channel

A survey of temporal filtering through the population of ON terminals is summarized in Figures 2C and 2D, and this revealed a striking difference with the OFF pathway. Although 83% of OFF terminals were activated by contrast, only 47% of 573 ON terminals responded similarly, i.e., with an increase in the average concentration of calcium above that measured at the same average intensity of steady light (Figure 2C). We also found

that 48% of ON terminals were *suppressed* by fluctuations in intensity, i.e., the average concentration of calcium fell below that measured under constant illumination of the same mean. The same pattern of activity was observed when applying the "reversed" frequency protocol, as shown in Figure S3. The frequency dependence of activation and suppression was very different. ON terminals activated by contrast displayed band-pass characteristics with peak activation at ~5 Hz, while contrast-suppressed terminals were tuned more flatly and broadly, being inhibited to a similar degree by frequencies ranging from 0.2 Hz to 11 Hz (Figure 2D, dotted line).

The responses of "contrast-suppressed" synapses can be compared to RGCs described as "uniformity detectors" in rabbits (Levick, 1967) or "suppressed-by-contrast" in cats (Rodieck, 1967). These RGCs maintain high rates of activity under spatially and temporally uniform illumination but are inhibited abruptly, and sometimes completely, by most forms of stimulation, including both light increments and decrements (de Monasterio, 1978; Mastronarde, 1985). Recent work in rabbits has demonstrated that both ON and OFF visual stimuli suppress the maintained firing of uniformity detectors primarily by the activation of transient and powerful inhibition from glycinergic synapses (Sivyer et al., 2010). The contrast-suppressed ON bipolar cells shown in Figures 1 and 2 are, by definition, activated by light increments, but a deviation from constant illumination appears to "flip" these to a less active state in which calcium levels fall to approximately the same as in darkness (Figures 1A and 2C), suggesting that the rate of vesicle release will be close to zero (Odermatt et al., 2012). One possible explanation is that the suppression of transmission originates in inhibitory inputs that these terminals receive within the IPL, and evidence for this idea was provided by pharmacological manipulation of inhibitory transmission (below and Figure 6).

Together, the results in Figures 1 and 2 demonstrate that low-frequency signals are transmitted to the IPL in two basic ways: inhibition of a specific sub-population of ON terminals and activation of OFF terminals.

Sustained OFF Signals Are Generated by Crossover Inhibition

Having identified different temporal channels for transmission of the visual signal to the IPL, we asked whether they could be distinguished anatomically. In zebrafish, bipolar cells transmit the visual signal through six strata (Figure 3A), and these outputs can be distinguished functionally by imaging and electrophysiology (Nikolaev et al., 2013; Dorostkar et al., 2010; Connaughton et al., 2004). A surprising but clear feature of these studies is that the ON and OFF outputs are not as cleanly segregated in the retina of zebrafish compared to other species (Masland, 2012). The histograms in Figures 3B and 3C show the distribution of the five frequency channels that we identified, separated into ON and OFF components. OFF terminals in Groups 1 and 2 were distributed similarly across the IPL, and both occurred at the highest density in layer 6 (Figure 3C; Layer 6 = 34/88 for Group 1 and 24/97 for Group 2; Layer 1 = 24/88 for Group 1 and 22/97 for Group 2). This distribution was notable because layer 6 also contained a high density of ON terminals activated by contrast (Figure 3B). The spatial distribution of OFF terminals tuned to higher frequencies (Group 3) was significantly different to those in Group 1 ($p < 0.02$ using Levene's test for non-normally distributed data and 20 equidistant bins through the IPL). Notably, the highest density of Group 3 terminals was found in Layer 1, which was almost devoid of ON terminals (Figures 3B and 3C).

Might the co-stratification of low-pass OFF terminals with ON terminals reflect a local role of crossover inhibition in generating sustained OFF responses? Experiments in which we blocked signal transmission through the ON pathway using L-AP4 (100 μM) confirmed that this was the case. Figure 3D shows how L-AP4 altered the distribution of cut-off frequencies across a population of 445 OFF terminals from 5 fish: the two distributions were found to be significantly different at $p < 10^{-5}$ using Levene's test. Terminals with low-pass properties (Group 1) were almost completely abolished, while the density of terminals with band-pass characteristics (Group 2) increased. Further, blocking the ON pathway reduced the gain of signaling through the OFF pathway across the range of frequencies tested (Figure 3E), supporting previous data from mice retina showing that crossover inhibition also acts to increase excitation of the OFF channel (Manookin et al., 2008).

Closer examination of the histograms in Figure 3D suggested that the action of L-AP4 might be specific for low-pass terminals, causing their conversion into terminals with band-pass characteristics. To test this idea more directly, we imaged individual terminals before and after injection of L-AP4 into the eye (Figure 4A; see also Figure S4). Examples of the effects on three terminals from Group 1 are shown in the upper part of Figure 4B: all were converted from low-pass to band-pass. In contrast, terminals characterized as Group 2 under control conditions retained these characteristics in the presence of L-AP4, and the same was found for terminals in Group 3 (Figures 4B and 4C). These results reveal a previously unrecognized role of crossover inhibition: the conversion of OFF synapses with band-pass characteristics into low-pass filters that will generate larger sustained responses to steps of light (Figure 2A, boxed area).

Glycinergic and GABAergic Control of Sustained Signals through the OFF Pathway

Most studies of crossover inhibition have identified glycinergic amacrine cells as the link between ON and OFF pathways (Wässle et al., 1998; Molnar and Werblin, 2007; Manookin et al., 2008; Hsueh et al., 2008). To investigate whether glycinergic inhibition is also involved in generating sustained responses, we blocked glycine receptors by intravitreal injection of strychnine at an estimated concentration of 5 μM (Figure 5A). Strychnine did not significantly affect the distribution of fc values when considering the complete population of OFF terminals (n = 216 OFF terminals from 5 fish; F test). However, inspection of the histograms in Figure 5A demonstrated that while the proportion of terminals in Group 3 remained constant, there was an increase in the fraction of terminals in Group 1 at the expense of terminals in Group 2. Limiting the comparison of the two distributions to terminals with cut-off frequencies below 6 Hz, they were found to be significantly different at the 5% level using an F test. This change could be expressed as the ratio of terminals in Group 1 versus Group 2, which increased from 1.37 in control conditions to 3.2 in

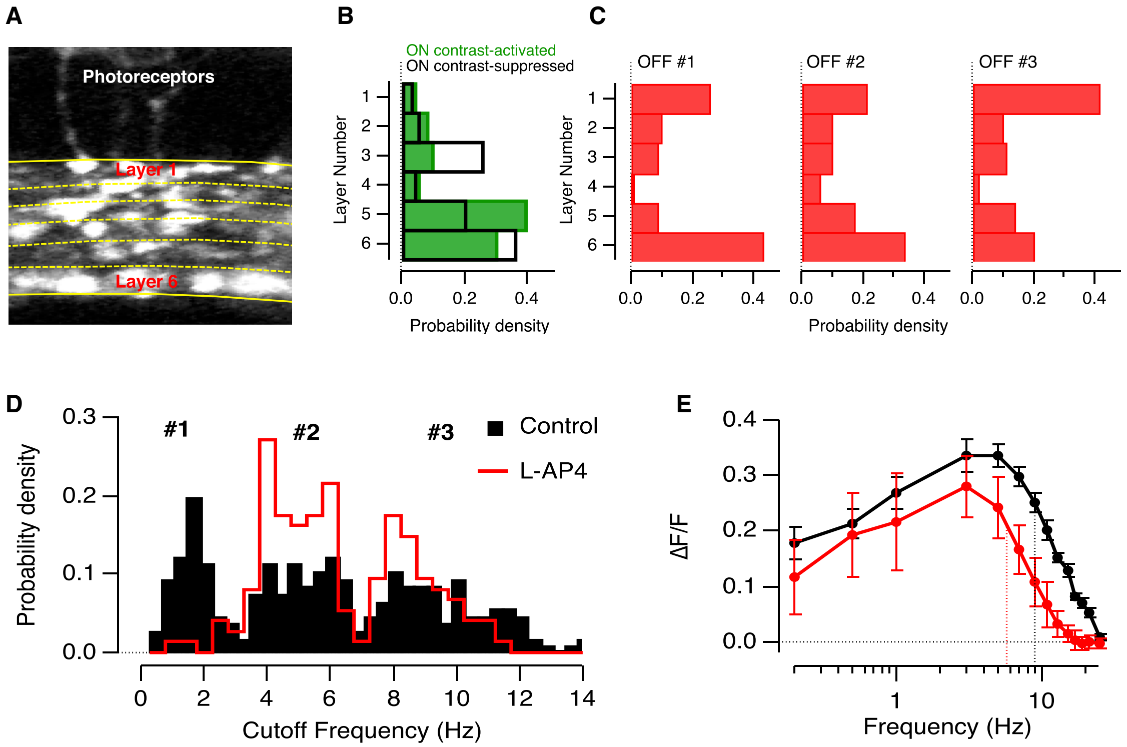

Figure 3. Sustained Responses in the OFF Channel Driven by Crossover Signals from the ON Channel

(A) View of the inner plexiform layer showing synaptic terminals of bipolar cells expressing SyGCaMP2. Yellow traces indicate the layers in the IPL. Field of view is 100 micrometers across.

(B) Spatial distribution of contrast-activated and contrast-suppressed ON bipolar terminals as a function of layer. The depth of the terminal in the IPL was measured from the photoreceptor side (layer 1) to ganglion cells (layer 6). Contrast-activated ON bipolar terminals showed the highest density in layer 5 and 6, whereas contrast-suppressed cells were mostly localized in layer 3 and 6.

(C) Spatial distribution of each OFF group as a function of layer. OFF terminals in Group 1 (low-pass) were at highest density in layer 6, whereas terminals in Group 3 (band-pass) were predominantly localized in layer 1. OFF bipolar terminals in Group 2 stratified throughout IPL with the highest density in layer 6.

(D) Histogram showing the distribution of cutoff frequencies (fc) in a population of 445 OFF terminals in 5 fish. Light transmission through ON pathway was inhibited by an intraocular injection of the mGluR6 agonist L-AP4 (100 μM estimated final concentration). Control is shown in black and L-AP4 in red. Note that OFF bipolar terminals in Group 1 (low-pass) are almost absent in presence of L-AP4.

(E) Plot of response amplitude as a function of frequency averaged across all OFF terminals, before (black trace) and after (red trace) L-AP4. Dashed lines represent the average cutoff frequency value (fc). Note that blocking signals through the ON pathway decreased the amplitude of responses in the OFF pathway across all range of frequencies.

See also Figure S4.

strychnine. These results indicate that glycinergic inhibition normally acts to convert a proportion of low-pass terminals in Group 1 into band-pass terminals in Group 2.

The action of glycinergic inhibition on bipolar cell synapses might be direct, through glycine receptors on the terminals, or indirect, through glycine receptors on GABAergic amacrine cells (Eggers and Lukasiewicz, 2011). If correct, the second possibility makes a strong prediction: blocking $GABA_A$ receptors should have the *opposite* effect to blocking glycine receptors and reduce the proportion of low-pass terminals in Group 1. This manipulation was made by introducing Gabazine at an estimated concentration of 10 μM, which caused a 73% decrease in the number of OFF terminals in Group 1 and a compensatory 60% increase in Groups 2 and 3 (Figure 5B; n = 248 OFF terminals from 5 fish). Gabazine and strychnine did not affect the temporal tuning curve of OFF groups (Figure S5). Together, the results in Figures 4 and 5 indicate that the generation of the sustained OFF channel depends almost exclusively on GABAergic inputs driven by ON bipolar cells, with these inputs themselves modulated by glycinergic amacrine cells. These observations are captured in the model shown in Figure 8.

Glycinergic and GABAergic Control of Signals through the ON Pathway

Inhibitory signaling within the IPL also regulated the temporal filters operating through the ON pathway. Blocking glycinergic inhibition with strychnine (5 μM) had three distinct effects: (1) the density of contrast-activated terminals was reduced by 60% (Figure 6A; $p < 0.05$); (2) the density of contrast-suppressed terminals was increased by 70% (Figure 6C, $p < 0.01$); and (3) in contrast-activated terminals, the gain of synaptic response at frequencies below ~3 Hz was significantly reduced, making the tuning curve sharper (Figure 6B). Under control conditions, the ratio of ON terminals suppressed by contrast versus

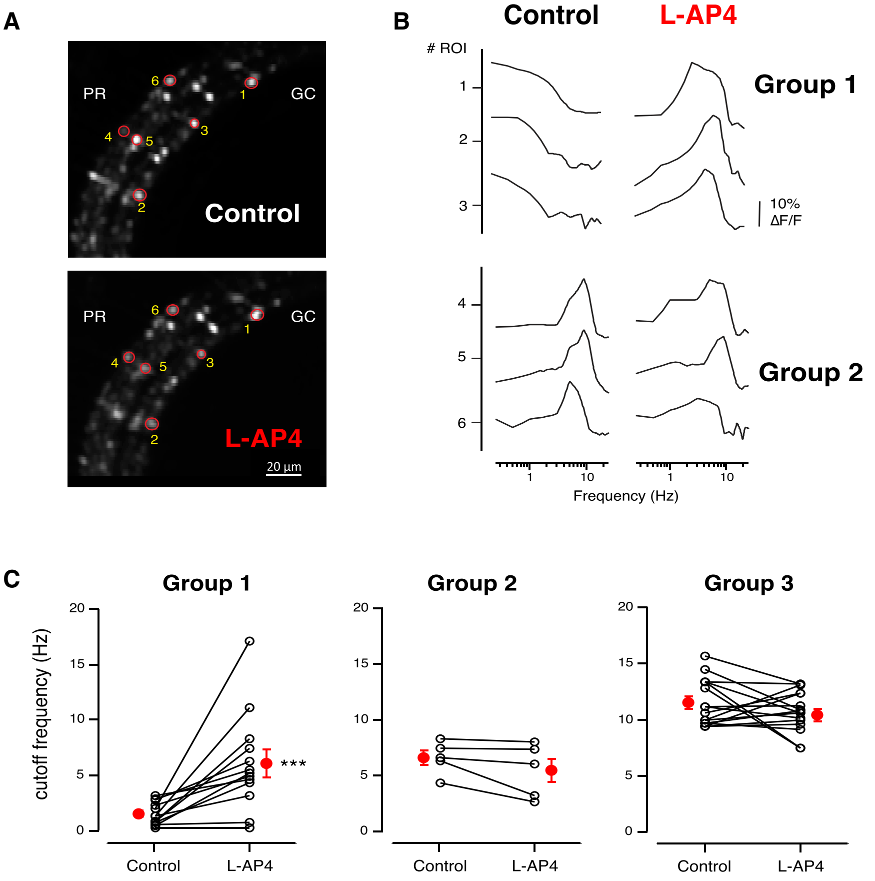

Figure 4. Crossover Inhibition Converts Band-Pass Terminals to Low-Pass

(A) A field of view showing the same population of bipolar cell terminals before and after the injection of L-AP4 into the eye of a zebrafish.

(B) Example of frequency tuning curves from three individual terminals from Group 1 (ROIs 1, 2, and 3 in A) and three from Group 2 (ROIs 4, 5, and 6) before and after L-AP4.

(C) Summary of the cutoff frequency values from all the OFF bipolar terminals in each group before and after L-AP4 (n = 34 terminals from 1 fish). Groups in control conditions were determined by K-means clustering (see Experimental Procedures). The cutoff frequency from the individual terminals was calculated as in Figure 1. Solid lines connect responses from the same terminals before and after L-AP4. Red dots represent mean ± SEM. ***p < 0.001.

activated by contrast was 1.3:1, and this ratio increased to 5.8:1 when glycinergic transmission was blocked.

Is the action of glycine direct or exerted through GABAergic amacrine cells? Blocking GABAergic transmission with Gabazine (10 μM) had an effect that was qualitatively opposite to strychnine, increasing the density of contrast-activated terminals by 90% (Figure 6A; $p < 0.01$). The ratio of ON terminals suppressed by contrast versus activated by contrast was reduced to 0.46:1 (Figure 6C), and the amplitude of these responses was significantly increased at lower frequencies (Figure 6D). Together, the results in Figure 6 indicate that ON terminals can switch between contrast-activated and contrast-suppressed modes of operation, with the balance determined by both GABAergic and glycinergic amacrine cells acting in a push-pull manner: GABAergic transmission pushes the population toward contrast suppression, while glycinergic transmission maintains the contrast-activated population. In the Discussion, we interpret these observations in terms of known patterns of connectivity in the IPL: ON terminals receive strong inhibitory input from contrast-activated GABAergic amacrine cells, which in turn experience strong lateral inhibition from glycinergic amacrine cells.

Band-Pass Filtering in Amacrine Cells

A notable feature of the effects of blocking glycine receptors was that the gain of transmission through the ON pathway was only reduced at frequencies below ~3 Hz (Figure 6B). Might this action reflect the frequency tuning of amacrine cells providing inhibition to bipolar cell synapses in the IPL? To investigate this possibility, we measured the temporal filters operating in amacrine cells using a zebrafish line expressing the calcium reporter SyGCaMP3 under the *ptf1a* promoter, which drives expression in all classes of amacrine cell (Jusuf and Harris, 2009; Nikolaev et al., 2013; Figure 7A). The resolution of our microscope did not allow us to distinguish all the processes belonging to individual amacrine cells, so we carried out a voxel-by-voxel analysis rather than attempting to segment the image into regions of interest defining individual processes (Nikolaou et al., 2012). Responses were measured across all voxels above a threshold intensity and then classified in two steps. First, by clustering of tuning curves using the k-means algorithm, which revealed two major types of synaptic tuning curve: "low" band-pass with peak transmission at about 5 Hz (Figure 7B), and "high" band-pass with peak transmission at 9–10 Hz (Figure 7C). Second, voxels were then separated further into ON (green), OFF (red), and ON-OFF (blue) according to their responses to step stimuli. Strikingly, amacrine cell responses tuned to lower and higher frequencies did not differ significantly in their position in the IPL (Figure 7D) and were found in ON, OFF, and ON-OFF subtypes. It may well be that further refinement of this functional classification will be possible by imaging retinae in which subsets of amacrine cells are labeled, allowing individual dendritic processes to be distinguished.

The stimulus frequencies that activated amacrine cells overlapped with those at which glycinergic inhibition modulated the ON pathway. For instance, Figure 6B shows that by isolating the glycinergic transmission by using Gabazine boosted signals at frequencies up to about 8 Hz. Notably, the sharp decline in amacrine cell activity at frequencies higher than 5 Hz was

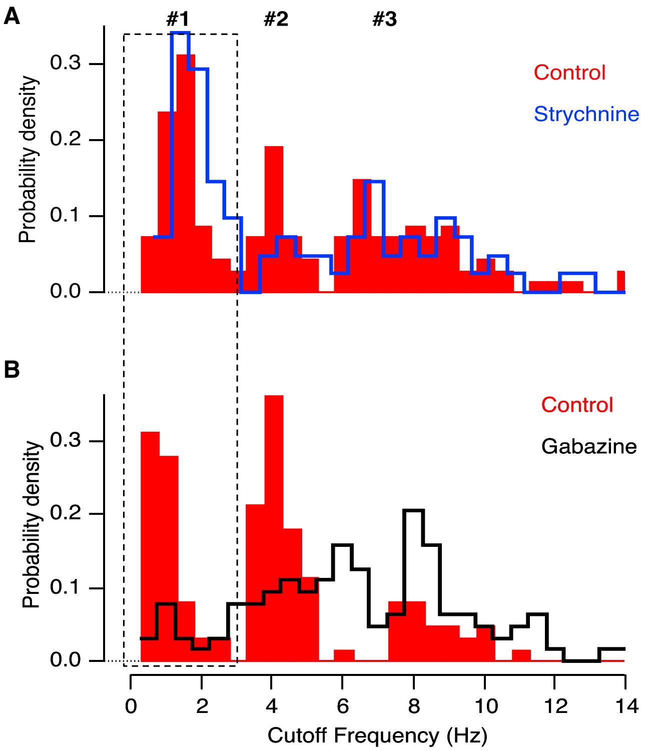

Figure 5. GABAergic Control of "Sustained" Signals through the OFF Pathway

(A) Histogram of the cutoff frequency (fc) of 230 OFF bipolar terminals from five fish before (control; red bars) and after (blue bars; 5 μM estimated final concentration) intraocular injection of strychnine. Strychnine increased the number of terminals in Group 1 by 30% (Group 1, n = 53 in control and n = 43 in strychnine; Group 2, n = 38 in control and n = 14 in strychnine; Group 3, n = 50 in control and n = 32 in strychnine).

(B) Histogram of the cutoff frequency (fc) of 248 OFF bipolar terminals from five fish before (control; red bars) and after (black bars; 10 μM estimated final concentration) intraocular injection of Gabazine. Gabazine reduced the number of terminals in Group 1 by 73% (Group 1, n = 44 in control and n = 12 in Gabazine; Group 2, n = 54 in control and n = 48 in Gabazine; Group 3, n = 23 in control and n = 73 in Gabazine).

See also Figure S5.

very similar to the decline observed through ON bipolar cells. The frequency tuning of the amacrine cell population was consistent with the idea that inhibition plays a key role in determining the gain of transmission through bipolar cells at low frequencies and, therefore, the establishment of the "sustained" pathway.

DISCUSSION

It has long been known that the output from the retina contains at least two distinct temporal channels—transient and sustained. The origin of the transient channel can be traced to cones providing the input to the retinal circuit, which generate oscillatory responses to flashes of light and act as band-pass filters (Schnapf et al., 1990). These transfer characteristics are maintained by the kinetic properties of glutamate receptors at the bipolar cell dendrites (Borghuis et al., 2014; DeVries, 2000; Puthussery et al., 2014) so that band-pass responses are also observed in the soma of bipolar cells (Baden et al., 2011; Burkhardt et al., 2007; Umino et al., 2008; DeVries, 2000). It has been less clear how the sustained channel is established within the inner retina. This study reveals that the first neural compartment to be specifically tuned to low frequencies is the synaptic terminal of OFF bipolar cells (Figures 1 and 2) and that the key circuit motif involves crossover inhibition from the ON pathway mediated through GABAergic amacrine cells. We also uncovered a previously unsuspected system by which the ON pathway signals sustained inputs: a suppression of synaptic activity in response to temporal modulations in light intensity, functionally analogous to RGCs acting as "uniformity detectors" (Mastronarde, 1985; Sivyer et al., 2010; Figures 1 and 2). Contrast suppression in bipolar cell synapses was modulated by glycinergic inhibition from amacrine cells (Figure 6).

A New Role of Crossover Inhibition

The function of crossover inhibition from ON to OFF pathways in the retina has been the subject of a number of studies (Demb and Singer, 2012; Lee et al., 2014; Buldyrev et al., 2012; Werblin, 2010; Pang et al., 2007; Molnar et al., 2009; Cafaro and Rieke, 2013), and here we propose a new function for this structural motif in the generation of a sustained temporal channel. A model of how this pathway operates based on our experimental findings is shown in Figure 8. The key feature is that lateral inhibition between glycinergic and GABAergic amacrine activated by the ON pathway acts on a subset of OFF bipolar cells terminals to convert their net synaptic output from band-pass to low-pass (Figure 8A). This model is based on two results. First, blocking the ON pathway with L-AP4 increased the proportion of band-pass synapses in the OFF pathway at the expense of low-pass (Figures 4C and 8B). Second, this effect was mimicked by Gabazine (Figures 5B and 8B), supporting previous findings that GABA-A receptors modulate lateral connections between amacrine cells (Eggers and Lukasiewicz, 2011). It is well established that GABAergic amacrine cells are themselves inhibited by glycinergic amacrine cells (Baccus, 2007; Masland, 2012), so this model predicts that blocking glycinergic transmission will result in the opposite effect—an increase in the number of low-pass synapses in the OFF pathway at the expense of band-pass (Figure 8C)—and this was observed experimentally (Figure 5A).

An alternative way of describing this model is that all OFF bipolar cells intrinsically respond as band-pass filters but that crossover inhibition can "sculpt" the tuning properties of the synaptic compartment to generate a low-pass output. Electrophysiological recording from the soma demonstrates that almost all bipolar cells in the zebrafish retina do indeed respond as band-pass filters (Baden et al., 2011). The soma is then separated from the synaptic terminal by a long thin axon of high resistance and a number of different conductances are localized to the synaptic compartment, including calcium channels and calcium-activated potassium channels that generate spikes (Burrone and Lagnado, 1997; Protti et al., 2000; Baden et al., 2011, 2014), and chloride conductances activated by

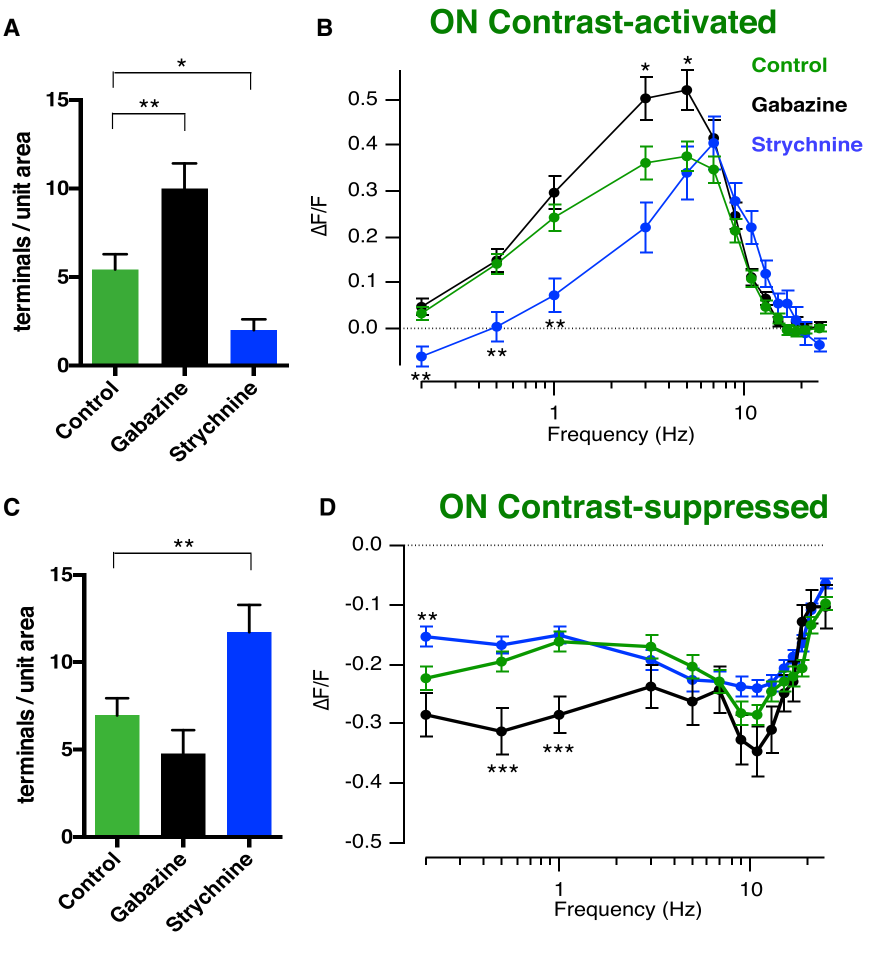

Figure 6. Glycinergic Control of "Sustained" Signals through the ON Pathway

(A) Density of contrast-activated ON terminals across the whole IPL, before and after injection of strychnine and Gabazine. Gabazine injection significantly increased the number of ON terminals generating a significant response at any frequency, while strychnine injection decreased it (**$p < 0.01$; *$p < 0.05$). Collected results from 632 ON terminals in 7 fish.

(B) Response amplitude as a function of frequency averaged from contrast-activated ON terminals before (green, n = 114) and after Gabazine (black, n = 120) or strychnine (blue, n = 28). Note that Gabazine increased peak gain, while strychnine reduced the response amplitudes at low frequencies.

(C) Density of contrast-suppressed ON terminals, before and after injection of strychnine and Gabazine. Strychnine significantly increased the number of contrast-suppressed terminals.

(D) Response amplitude as a function of frequency averaged from contrast-suppressed ON terminals before (green, n = 146) and after Gabazine (black, n = 57) or strychnine (blue, n = 164).

GABA or glycine that directly modulate glutamate release (Borghuis et al., 2014). In the future, it will be important to identify the biophysical mechanisms by which GABAergic feedback damps high-frequency signals and/or amplifies low-frequency components.

Potential Mechanisms of Suppression by Contrast

Ganglion cells suppressed by temporal contrast, also known as "uniformity detectors" have been recognized in a number of species and decrease their firing rate in response to changes in the visual scene (Mastronarde, 1985; de Monasterio, 1978; Sivyer et al., 2010; Cleland and Levick, 1974; Hoshi et al., 2013; Caldwell et al., 1978; Levick, 1967). We are not aware that contrast suppression has been observed electrophysiologically in bipolar cells but nonetheless found a large population of ON terminals that were strongly inhibited by fluctuations across all frequencies above ~0.2 Hz (Figures 1, 2, 6C, and 6D). It seems likely that excitatory synaptic inputs with these functional characteristics will contribute to building RGCs acting as uniformity detectors.

The simplest mechanism by which temporal contrast would deactivate bipolar cell synapses is hyperpolarization, but this has not been observed by making recordings at the cell body (Baden et al., 2011). It therefore seems likely that contrast acts to suppress ON terminals locally, by activating inhibitory inputs that "flip" the synaptic compartment from an active state into one that is hyperpolarized below the threshold for activation of calcium channels. This idea is supported by studies demonstrating that the synaptic compartment of ON bipolar cell is "bi-stable" and can jump from a depolarized state in which voltage-sensitive calcium channels are open and the synapse is tonically active into one in which calcium channels are closed (Burrone and Lagnado, 1997). Such "flips" between active and inactive states can be caused by small injections of current around the threshold for activation of calcium channels (Baden et al., 2011). Further, the highest density of contrast-suppressed bipolar cell terminals was observed in the deeper layers of the IPL (Figure 3), where bistability has been previously observed in ON bipolar cells isolated from goldfish (Burrone et al., 2002). The switching off of synaptic activity can be thought of as another route to signal the presence of low-frequency fluctuations in the visual input.

Recent work from a number of laboratories has reinforced the idea that individual bipolar cells do not necessarily reflect a single filter or channel in the transformation of the visual signal (Asari and Meister, 2012; Baden et al., 2014). Many bipolar cells deliver their output through multiple synaptic compartments, and these are key sites of signal integration that are at least partially isolated from each other and the soma (Masland, 2012; Baden et al., 2011). In this study, we have demonstrated how the inhibitory signals received by these synaptic compartments provide the origin for two functional channels that can be subsequently recognized in the retinal output: the

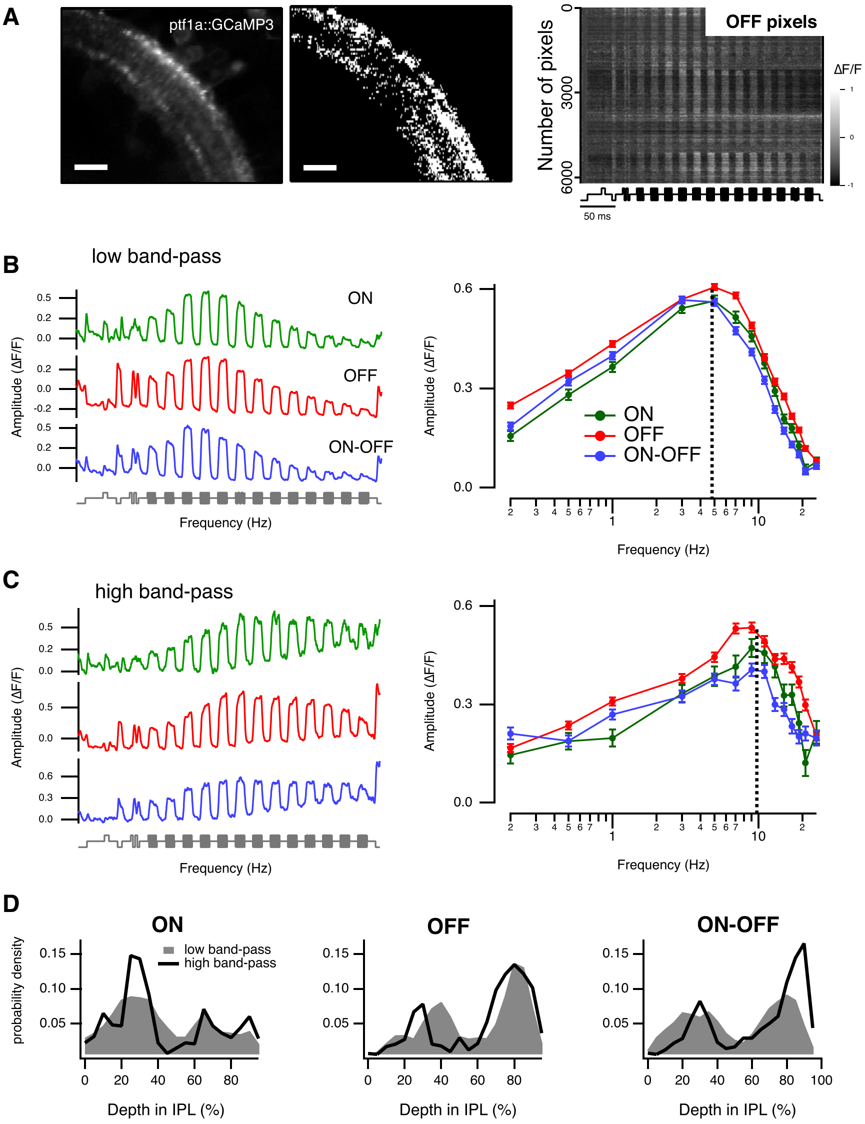

Figure 7. Voxel-Based Analysis of Calcium Signal in Amacrine Cells Reveals Diversity in Temporal Tuning

(A) Left: view of the IPL showing amacrine cells expressing SyGCaMP3 (left) and the respective pixel-mask (right). Scale bar represents 20 μm. Right: raster plot showing the relative change in fluorescence for 6,210 pixels during a "forward" frequency seep. Only OFF voxels are shown, as defined by the responses to steps of light.

(B and C) K-means clustering revealed two major types of temporal tuning in amacrine cells, "low" band-pass (B, peak transmission at 4.6 ± 0.2 Hz and fc = 9.8 ± 0.11 Hz) and "high" band-pass (C, peak transmission at 9.9 ± 0.3 Hz and fc = 13.9 ± 0.2 Hz). Voxels were then separated further into ON (green), OFF (red), and ON-OFF (blue). Results were collected from five fish. The left-hand plots show averaged SyGCaMP3 responses of the three groups classified as low and high band-pass from a total of between 5,207 and 8,540 voxels from five fish. The right-hand plots show response amplitude as a function of frequency.

(D) Spatial distribution within the IPL of low band-pass (filled gray regions) and high band-pass (solid lines) voxels as a function of dendrite stratification in the IPL for ON (left), OFF (middle), and ON-OFF (right) pixels. Stratification is plotted such that 0% is the boundary with the ganglion cell layer and 100% the boundary with the inner nuclear layer.

"sustained" channel and "uniformity detectors." It seems likely that our understanding of the retinal circuit will continue to advance by considering individual synapses as distinct functional units rather than simple relays of signals observed in the cell body.

EXPERIMENTAL PROCEDURES

Animals

We used transgenic zebrafish (*Danio rerio*) maintained on a 14 hr:10 hr light/dark cycle at 28°C (Nüsslein-Volhard and Dahm, 2002). The Home Office of the UK, the Medical Research Council Laboratory of Molecular Biology Ethical Review Committee, and the University of Sussex Ethical Review Committee approved all procedures for animal maintenance and imaging. A total of 33 fish were used in these experiments. To image calcium signals in the synaptic terminals of bipolar cells, we used the synaptically localized calcium reporter SyGCaMP2 under the RibeyeA promoter. In the line of fish we used (*Tg(–1.8ctbp2:SyGCaMP2)lmb*), expression of SyGCaMP2 within the inner plexiform layer only occurs in bipolar cell terminals (Odermatt et al., 2012; Esposti et al., 2013; Dreosti et al., 2011; Baden et al., 2011). To image synaptic calcium signals in amacrine cells, we used *ptf1a:gal4;UAS:SyGCaMP3* fish. The *ptf1a* promoter drives expression across all types of amacrine cell but not bipolar cells (Jusuf and Harris, 2009; Nikolaev et al., 2013).

It should be noted that SyGCaMP2 does not respond instantaneously to a change in calcium concentration: the off (unbinding) time constant is about 300 ms (Dreosti et al., 2009), so the fluorescence signal can be thought of as a low-pass version of the underlying calcium signal. The on time constant is significantly shorter, about 20 ms, and introduces less of a distortion (Tallini et al., 2006).

In Vivo Multi-photon Imaging

Zebrafish larvae (7–10 days post-fertilization) were immobilized in 2.5% low melting point agarose (Biogene) in E2 medium on a glass coverslip (0 thickness) and mounted in a chamber where they were superfused with E2, as described previously (Odermatt et al., 2012). To prevent eye movements, the ocular muscles were paralyzed by injection of 1 nL of α-bungarotoxin (2 mg/mL) behind the eye. Imaging experiments were performed in the afternoon (2–8 p.m., 7–13 hr after light onset). Fish larvae were kept in E2 medium containing 1-phenyl-2-thiourea (200 μM, Sigma Aldrich) from 28 hr post-fertilization to minimize pigmentation.

Imaging of bipolar cell terminals and amacrine cells was carried out using a custom-built two-photon microscope equipped with a mode-locked titanium-sapphire laser (Chameleon, Coherent) tuned to 915 nm and an Olympus LUMPlanFI 403 water immersion objective (NA 0.8). Fluorescence emission was captured both by the objective and a substage oil condenser (Olympus), filtered through GFP emission filters (HQ 535/50, Chroma Technology) before detection with photomultiplier tubes (Hamamatsu). Scanning and image

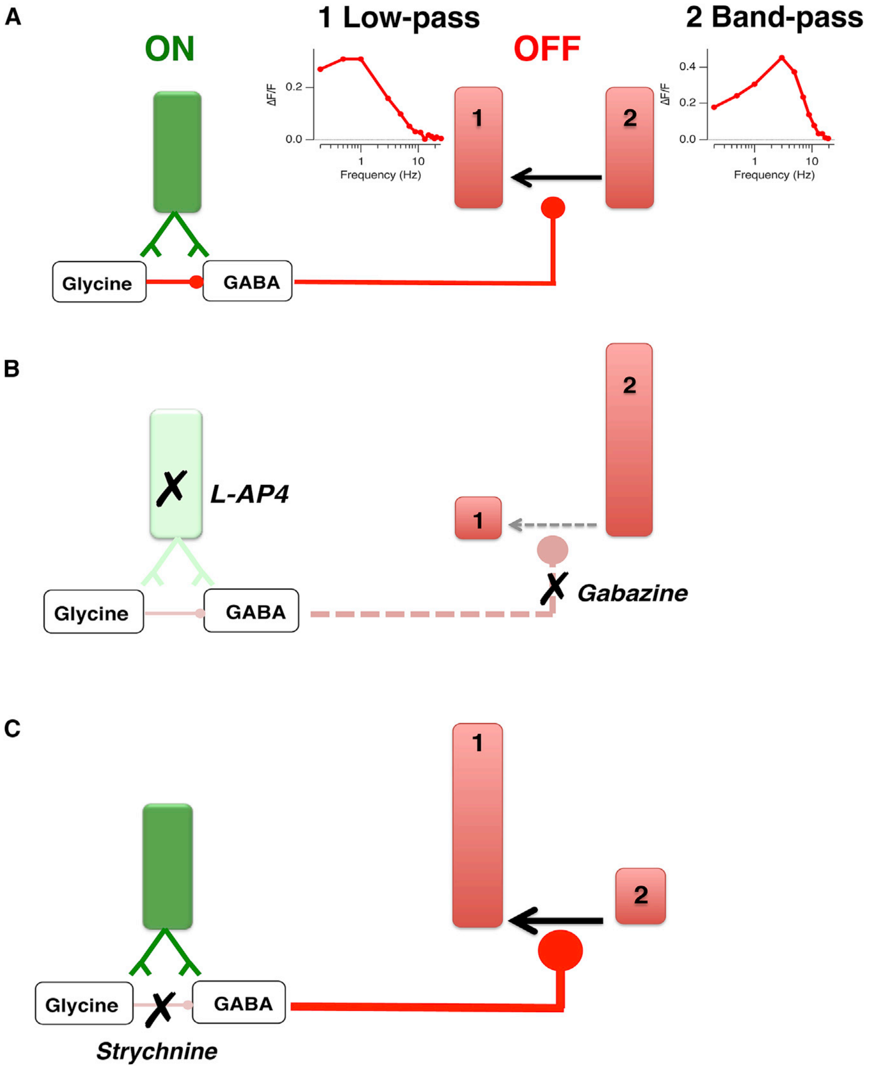

Figure 8. Schematic of the Synaptic Mechanism Controlling the Sustained OFF Channel in the IPL

(A) During light stimulation, GABAergic and glycinergic amacrine cells (ACs) receive excitatory glutamatergic inputs from ON bipolar cells. The activated GABAergic ACs synapse onto OFF bipolar terminals to shape the temporal properties of the OFF channel. Transient/band-pass responses become sustained/low-pass through the action of the GABAergic ACs. The amount of inhibition through this crossover mechanism is controlled by a lateral connection with the activated glycinergic AC. Under control conditions, both sustained and transient channels are balanced and contribute almost equally to the temporal properties of the OFF channel.

(B) OFF bipolar terminals dramatically change their temporal output when signals arriving from the ON pathway are blocked. Using either L-AP4 to inhibit the activation of the ON pathway or Gabazine to block the synaptic transmission through the GABAergic ACs almost abolishes OFF terminals responding as low-pass filters and increases the number of OFF terminals responding as band-pass filters.

(C) The role of the lateral synapse between glycinergic and GABAergic ACs is evident in the presence of strychnine. GABAergic ACs are relieved from glycinergic signaling by strychnine, which results in an augmented inhibition onto the terminals of OFF bipolar cells and thus an increase in the number of terminals responding as low-pass filters.

acquisition were controlled under ScanImage v.3.6 software (Pologruto et al., 2003). Image sequences were typically acquired at 10 Hz (256 × 100 pixels per frame, 1 ms per line).

Light Stimulation and Drug Application

Wide-field light stimuli were generated by an amber LED (lmax = 590 nm, Phillips Luxeon, 350 mA, 3 V), filtered through a 590/10 nm BP filter (Thorlabs), and delivered through a light guide placed close to the eye of the fish. These wavelengths will stimulate L-cones about 100× more effectively than M-cones (Endeman et al., 2013), although some weaker stimulation of rod pathways might also be expected (Li et al., 2012). Stimulation was synchronized to image acquisition through Igor Pro software (Wavemetrics). The mean intensity of the stimulus was controlled by neutral density filters to 55 nW/mm^2 (maximum light intensity, 110 nW/mm^2, equivalent to 3.3 × 10^{11} photons/mm^2 s^{-1}) and modulations around this mean were generated by a custom-built LED driver that switched the driving current at 10 kHz while adjusting the duty cycle. Frequency tuning was assessed by stimulating the dark-adapted fish with a series of 10 s square wave light oscillations around a constant light level at 90% contrast at 14 different frequencies, ranging from 0.2 to 25 Hz (Esposti et al., 2013). Please note that the dynamics of our reporter, SyGCaMP2, does not allow any quantitative evaluation of the behavior over 10 Hz as previously described in the zebrafish retina (Esposti et al., 2013).

Pharmacological manipulation was achieved by injection of substances diluted in oxygenated fish Ames' solution (Sigma Aldrich) into the eye, as described by (Esposti et al., 2013). Final concentrations in the extracellular space were estimated as 100 μM for the selective agonist for the group III metabotropic glutamate receptors L-AP4 (Tocris), 10 μM for the $GABA_A$ receptor antagonist Gabazine (Tocris), and 5 μM for the glycine receptor antagonist strychnine (Sigma). Injection of Ames' solution alone did not have an effect on the frequency responses (data not shown).

Image Analysis

Image sequences were analyzed using SARFIA, a set of custom-written procedures for IgorPro (Dorostkar et al., 2010). Regions of interest (ROIs) defining bipolar cell terminals and amacrine cells were defined by thresholding the Laplacian Transform of an averaged image. If necessary, images were registered to correct for small movements in the x and y directions. Image sequences showing large movements, especially in the z direction, were rejected.

ON and OFF cells were defined by their responses to steps of light (as in the first 50 s of the protocol shown in Figure 1) and contrast-enhanced and contrast-suppressed cells were distinguished by their response to oscillations around the mean light level. To analyze the response at a given frequency, we measured the change in fluorescence during modulation of light intensity relative to the baseline measured in the preceding 5 s of steady light. Tuning curves were then constructed by repeating these measurements over a range of frequencies.

To reveal functional subtypes of cells, we clustered responses over a range of frequencies using the K-means algorithm in Multiexperiment Viewer Software (http://www.tm4.org/mev.html), with the number of clusters chosen

based on the figure of merit, which calculates the minimum number that provided the largest improvement in performance. Prior to clustering, traces from individual cells were normalized so that only the dynamics of the response (rather than the amplitude) determined separation.

All errors indicated in the text and shown in the figures represent SEM.

SUPPLEMENTAL INFORMATION

Supplemental Information includes five figures and can be found with this article online at http://dx.doi.org/10.1016/j.neuron.2016.03.015.

AUTHOR CONTRIBUTIONS

J.M.R., S.R., and L.L. designed the study. J.M.R., S.R., and H.D. carried out the experiments. J.M.R., S.R., H.D., and L.L. carried out the analysis. J.M.R., S.R., and L.L. wrote the manuscript.

ACKNOWLEDGMENTS

This work was supported by grants from Wellcome Trust (Grant 102905) and BBSRC (BB/L021528/1) to L.L., and an E.U. Marie Curie Intra-European Fellowship to J.M.R. (2010-IEF-272823 FSES). We would like to thank all of the members of the L.L. laboratory for discussions that contributed to this work.

Received: April 13, 2015
Revised: January 21, 2016
Accepted: March 2, 2016
Published: April 7, 2016

REFERENCES

Asari, H., and Meister, M. (2012). Divergence of visual channels in the inner retina. Nat. Neurosci. *15*, 1581–1589.

Awatramani, G.B., and Slaughter, M.M. (2000). Origin of transient and sustained responses in ganglion cells of the retina. J. Neurosci. *20*, 7087–7095.

Baccus, S.A. (2007). Timing and computation in inner retinal circuitry. Annu. Rev. Physiol. *69*, 271–290.

Baden, T., Esposti, F., Nikolaev, A., and Lagnado, L. (2011). Spikes in retinal bipolar cells phase-lock to visual stimuli with millisecond precision. Curr. Biol. *21*, 1859–1869.

Baden, T., Nikolaev, A., Esposti, F., Dreosti, E., Odermatt, B., and Lagnado, L. (2014). A synaptic mechanism for temporal filtering of visual signals. PLoS Biol. *12*, e1001972.

Borghuis, B.G., Looger, L.L., Tomita, S., and Demb, J.B. (2014). Kainate receptors mediate signaling in both transient and sustained OFF bipolar cell pathways in mouse retina. J. Neurosci. *34*, 6128–6139.

Buldyrev, I., Puthussery, T., and Taylor, W.R. (2012). Synaptic pathways that shape the excitatory drive in an OFF retinal ganglion cell. J. Neurophysiol. *107*, 1795–1807.

Burkhardt, D.A., Fahey, P.K., and Sikora, M.A. (2007). Retinal bipolar cells: temporal filtering of signals from cone photoreceptors. Vis. Neurosci. *24*, 765–774.

Burrone, J., and Lagnado, L. (1997). Electrical resonance and Ca2+ influx in the synaptic terminal of depolarizing bipolar cells from the goldfish retina. J. Physiol. *505*, 571–584.

Burrone, J., Neves, G., Gomis, A., Cooke, A., and Lagnado, L. (2002). Endogenous calcium buffers regulate fast exocytosis in the synaptic terminal of retinal bipolar cells. Neuron *33*, 101–112.

Cafaro, J., and Rieke, F. (2013). Regulation of spatial selectivity by crossover inhibition. J. Neurosci. *33*, 6310–6320.

Caldwell, J.H., Daw, N.W., and Wyatt, H.J. (1978). Effects of picrotoxin and strychnine on rabbit retinal ganglion cells: lateral interactions for cells with more complex receptive fields. J. Physiol. *276*, 277–298.

Cleland, B.G., and Levick, W.R. (1974). Properties of rarely encountered types of ganglion cells in the cat's retina and an overall classification. J. Physiol. *240*, 457–492.

Connaughton, V.P., Graham, D., and Nelson, R. (2004). Identification and morphological classification of horizontal, bipolar, and amacrine cells within the zebrafish retina. J. Comp. Neurol. *477*, 371–385.

de Monasterio, F.M. (1978). Properties of ganglion cells with atypical receptive-field organization in retina of macaques. J. Neurophysiol. *41*, 1435–1449.

Demb, J.B., and Singer, J.H. (2012). Intrinsic properties and functional circuitry of the AII amacrine cell. Vis. Neurosci. *29*, 51–60.

DeVries, S.H. (2000). Bipolar cells use kainate and AMPA receptors to filter visual information into separate channels. Neuron *28*, 847–856.

Dorostkar, M.M., Dreosti, E., Odermatt, B., and Lagnado, L. (2010). Computational processing of optical measurements of neuronal and synaptic activity in networks. J. Neurosci. Methods *188*, 141–150.

Dreosti, E., and Lagnado, L. (2011). Optical reporters of synaptic activity in neural circuits. Exp. Physiol. *96*, 4–12.

Dreosti, E., Odermatt, B., Dorostkar, M.M., and Lagnado, L. (2009). A genetically encoded reporter of synaptic activity in vivo. Nat. Methods *6*, 883–889.

Dreosti, E., Esposti, F., Baden, T., and Lagnado, L. (2011). In vivo evidence that retinal bipolar cells generate spikes modulated by light. Nat. Neurosci. *14*, 951–952.

Eggers, E.D., and Lukasiewicz, P.D. (2011). Multiple pathways of inhibition shape bipolar cell responses in the retina. Vis. Neurosci. *28*, 95–108.

Endeman, D., Klaassen, L.J., and Kamermans, M. (2013). Action spectra of zebrafish cone photoreceptors. PLoS ONE *8*, e68540.

Esposti, F., Johnston, J., Rosa, J.M., Leung, K.-M., and Lagnado, L. (2013). Olfactory stimulation selectively modulates the OFF pathway in the retina of zebrafish. Neuron *79*, 97–110.

Hoshi, H., Tian, L.-M., Massey, S.C., and Mills, S.L. (2013). Properties of the ON bistratified ganglion cell in the rabbit retina. J. Comp. Neurol. *521*, 1497–1509.

Hsueh, H.-A., Molnar, A., and Werblin, F.S. (2008). Amacrine-to-amacrine cell inhibition in the rabbit retina. J. Neurophysiol. *100*, 2077–2088.

Jusuf, P.R., and Harris, W.A. (2009). Ptf1a is expressed transiently in all types of amacrine cells in the embryonic zebrafish retina. Neural Dev. *4*, 34.

Lee, S., Chen, L., Chen, M., Ye, M., Seal, R.P., and Zhou, Z.J. (2014). An unconventional glutamatergic circuit in the retina formed by vGluT3 amacrine cells. Neuron *84*, 708–715.

Levick, W.R. (1967). Receptive fields and trigger features of ganglion cells in the visual streak of the rabbits retina. J. Physiol. *188*, 285–307.

Li, Y.N., Tsujimura, T., Kawamura, S., and Dowling, J.E. (2012). Bipolar cell-photoreceptor connectivity in the zebrafish (Danio rerio) retina. J. Comp. Neurol. *520*, 3786–3802.

Manookin, M.B., Beaudoin, D.L., Ernst, Z.R., Flagel, L.J., and Demb, J.B. (2008). Disinhibition combines with excitation to extend the operating range of the OFF visual pathway in daylight. J. Neurosci. *28*, 4136–4150.

Masland, R.H. (2001). The fundamental plan of the retina. Nat. Neurosci. *4*, 877–886.

Masland, R.H. (2012). The neuronal organization of the retina. Neuron *76*, 266–280.

Mastronarde, D.N. (1985). Two types of cat retinal ganglion cells that are suppressed by contrast. Vision Res. *25*, 1195–1196.

Molnar, A., and Werblin, F. (2007). Inhibitory feedback shapes bipolar cell responses in the rabbit retina. J. Neurophysiol. *98*, 3423–3435.

Molnar, A., Hsueh, H.-A., Roska, B., and Werblin, F.S. (2009). Crossover inhibition in the retina: circuitry that compensates for nonlinear rectifying synaptic transmission. J. Comput. Neurosci. *27*, 569–590.

Nikolaev, A., Leung, K.-M., Odermatt, B., and Lagnado, L. (2013). Synaptic mechanisms of adaptation and sensitization in the retina. Nat. Neurosci. *16*, 934–941.

Nikolaou, N., Lowe, A.S., Walker, A.S., Abbas, F., Hunter, P.R., Thompson, I.D., and Meyer, M.P. (2012). Parametric functional maps of visual inputs to the tectum. Neuron *76*, 317–324.

Nüsslein-Volhard, C., and Dahm, R. (2002). Zebrafish: A Practical Approach (Oxford University Press).

Odermatt, B., Nikolaev, A., and Lagnado, L. (2012). Encoding of luminance and contrast by linear and nonlinear synapses in the retina. Neuron *73*, 758–773.

Pang, J.-J., Gao, F., and Wu, S.M. (2007). Cross-talk between ON and OFF channels in the salamander retina: indirect bipolar cell inputs to ON-OFF ganglion cells. Vision Res. *47*, 384–392.

Pologruto, T.A., Sabatini, B.L., and Svoboda, K. (2003). ScanImage: flexible software for operating laser scanning microscopes. Biomed. Eng. Online *2*, 13.

Protti, D.A., Flores-Herr, N., and von Gersdorff, H. (2000). Light evokes Ca2+ spikes in the axon terminal of a retinal bipolar cell. Neuron *25*, 215–227.

Puthussery, T., Percival, K.A., Venkataramani, S., Gayet-Primo, J., Grünert, U., and Taylor, W.R. (2014). Kainate receptors mediate synaptic input to transient and sustained OFF visual pathways in primate retina. J. Neurosci. *34*, 7611–7621.

Rodieck, R.W. (1967). Receptive fields in the cat retina: a new type. Science *157*, 90–92.

Roska, B., and Werblin, F. (2001). Vertical interactions across ten parallel, stacked representations in the mammalian retina. Nature *410*, 583–587.

Schnapf, J.L., Nunn, B.J., Meister, M., and Baylor, D.A. (1990). Visual transduction in cones of the monkey Macaca fascicularis. J. Physiol. *427*, 681–713.

Sivyer, B., Taylor, W.R., and Vaney, D.I. (2010). Uniformity detector retinal ganglion cells fire complex spikes and receive only light-evoked inhibition. Proc. Natl. Acad. Sci. USA *107*, 5628–5633.

Tallini, Y.N., Ohkura, M., Choi, B.-R., Ji, G., Imoto, K., Doran, R., Lee, J., Plan, P., Wilson, J., Xin, H.-B., et al. (2006). Imaging cellular signals in the heart in vivo: Cardiac expression of the high-signal Ca2+ indicator GCaMP2. Proc. Natl. Acad. Sci. USA *103*, 4753–4758.

Umino, Y., Solessio, E., and Barlow, R.B. (2008). Speed, spatial, and temporal tuning of rod and cone vision in mouse. J. Neurosci. *28*, 189–198.

Wässle, H. (2004). Parallel processing in the mammalian retina. Nat. Rev. Neurosci. *5*, 747–757.

Wässle, H., Koulen, P., Brandstätter, J.H., Fletcher, E.L., and Becker, C.-M. (1998). Glycine and GABA receptors in the mammalian retina. Vision Res. *38*, 1411–1430.

Werblin, F.S. (2010). Six different roles for crossover inhibition in the retina: correcting the nonlinearities of synaptic transmission. Vis. Neurosci. *27*, 1–8.

Werblin, F.S. (2011). The retinal hypercircuit: a repeating synaptic interactive motif underlying visual function. J. Physiol. *589*, 3691–3702.

Neuron, Volume *90*

Supplemental Information

Crossover Inhibition Generates Sustained Visual Responses in the Inner Retina

Juliana M. Rosa, Sabine Ruehle, Huayu Ding, and Leon Lagnado

Crossover-inhibition generates sustained visual responses in the inner retina

Supplementary Figure Legends

Figure S1: Two further functional classes of bipolar cell terminal. Related to Figures 1 and 2.

(A and B). Although the majority of bipolar cell terminals fell into one of the five functional classes shown in Figs. 1 and 2, we also observed two further small but distinct populations: OFF terminals that were suppressed-by-contrast (4%, Fig. S1A; 37 terminals from 7 fish) and others that could not be classified as ON or OFF but displayed band-pass characteristics (Fig. S1B; 13 terminals from 7 fish). *Top*: Raster plot showing the relative changes in fluorescence (ΔF/F). *Bottom*: Averaged SyGCaMP2 response from the same contrast-suppressed and frequency-activated OFF terminals shown in raster plots. SEM indicated in gray. (C) Plot of response amplitude as a function of frequency averaged from the same population of contrast-suppressed OFF terminals (black) and unclassified band-pass terminals (blue; fc = 15.1 ± 1.5 Hz).

Figure S2: The three temporal channels through the OFF pathway are not an artifact generated by activity-dependent adaptation. Related to Figure 1 and 2.

Application of a "reversed" (i.e. from highest to lowest frequency) stimulation generates the same temporal tuning response in OFF terminals observed in Figure 1 and 2.

(A and B) Averaged responses within each of the 3 groups to stimuli of frequency indicated. 94 OFF terminals were sorted into 3 distinct groups according to the K-means clustering shown in B. (C) Plot of response amplitude as a function of frequency for the three groups of OFF terminals shown in A and B. (D) Spatial distribution of each OFF group as a function of layer. OFF terminals in Group 1 (low-pass) were at highest density in layer 6, whereas terminals in Group 3 (band-pass) were predominantly localized in layer 1. OFF bipolar terminals in Group 2 stratified throughout IPL.

Figure S3: The two temporal channels through the ON pathway are not an artifact generated by activity-dependent adaptation. Related to Figure 1 and 2.

Application of a “reversed” (i.e. from highest to lowest frequency) stimulation generates the same temporal tuning response in ON terminals observed in Figure 1 and 2.

(A) Averaged responses from 60 ON contrast-activated (green) and 70 ON contrast-suppressed (black) terminals. Light stimulation is shown in grey. (B) Plot of response amplitude as a function of frequency for the two groups of ON terminals shown in A. (C) Spatial distribution of contrast-activated and contrast-suppressed ON bipolar terminals as a function of layer. The depth of the terminal in the IPL was measured from the photoreceptor side (layer 1) to ganglion cells (layer 6).

Figure S4: Effect of crossover inhibition according to layers of the IPL. Related to Figures 3 and 4.

(A) As shown in Fig. 3, layer 6 contained the highest density of sustained OFF terminals in Group 1 (low-pass) under control conditions: blocking ON signals with L-AP4 almost completely abolished these sustained responses while simultaneously increasing the

density of terminals in Group 2. This effect also manifested itself as an almost complete abolition of the response to a step of light (B) and a shift in the tuning curve averaged over all OFF terminals from low-pass to band-pass (C). Notably, none of these three effects was apparent in the responses of OFF terminals in Layer 1 (D), which is almost completely devoid of an ON input. These results reveal a previously unrecognized role of crossover inhibition: the conversion of OFF synapses with band-pass characteristics into low-pass filters.

Figure S5: Effects of glycinergic and GABAergic inhibition on the gain of transmission through the three major OFF temporal channels. Related to Figure 5.

(A) Plot of response amplitude as a function of frequency averaged over the same populations of OFF terminals shown in Figure 5A-B, before (red) and after gabazine (black) or strychnine (blue). No significant changes were detected in the amplitude and in the tuning of all three groups.

Figure S1

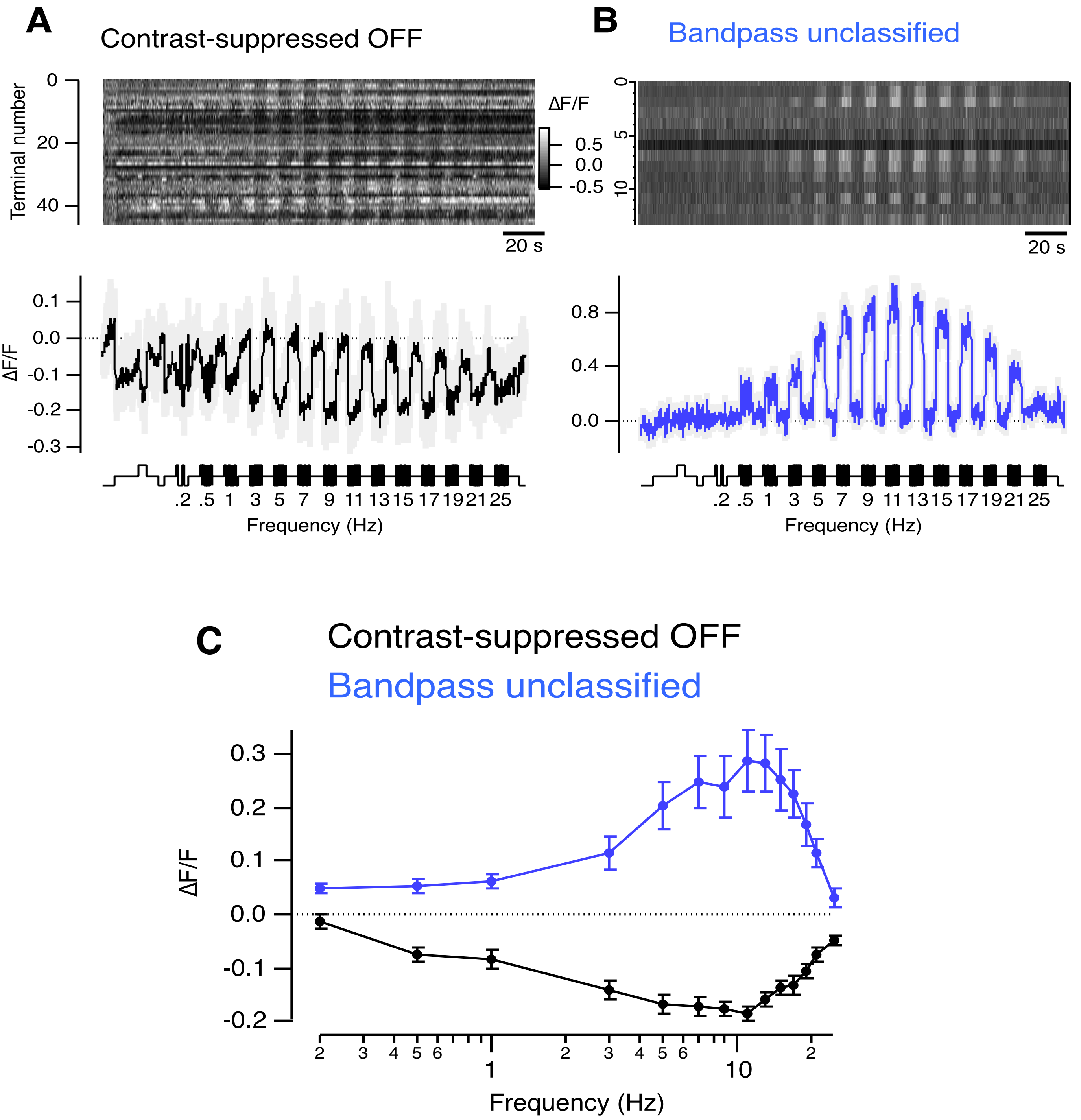
A
Contrast-suppressed OFF
Terminal number
0
20
40
ΔF/F
0.5
0.0
-0.5
20 s
B
Bandpass unclassified
0
5
10
20 s
ΔF/F
0.1
0.0
-0.1
-0.2
-0.3
0.8
0.4
0.0
.2 .5 1 3 5 7 9 11 13 15 17 19 21 25
Frequency (Hz)
.2 .5 1 3 5 7 9 11 13 15 17 19 21 25
Frequency (Hz)
C
Contrast-suppressed OFF
Bandpass unclassified
ΔF/F
0.3
0.2
0.1
0.0
-0.1
-0.2
2 3 4 5 6
1
2 3 4 5 6
10
2
Frequency (Hz)

Figure S2

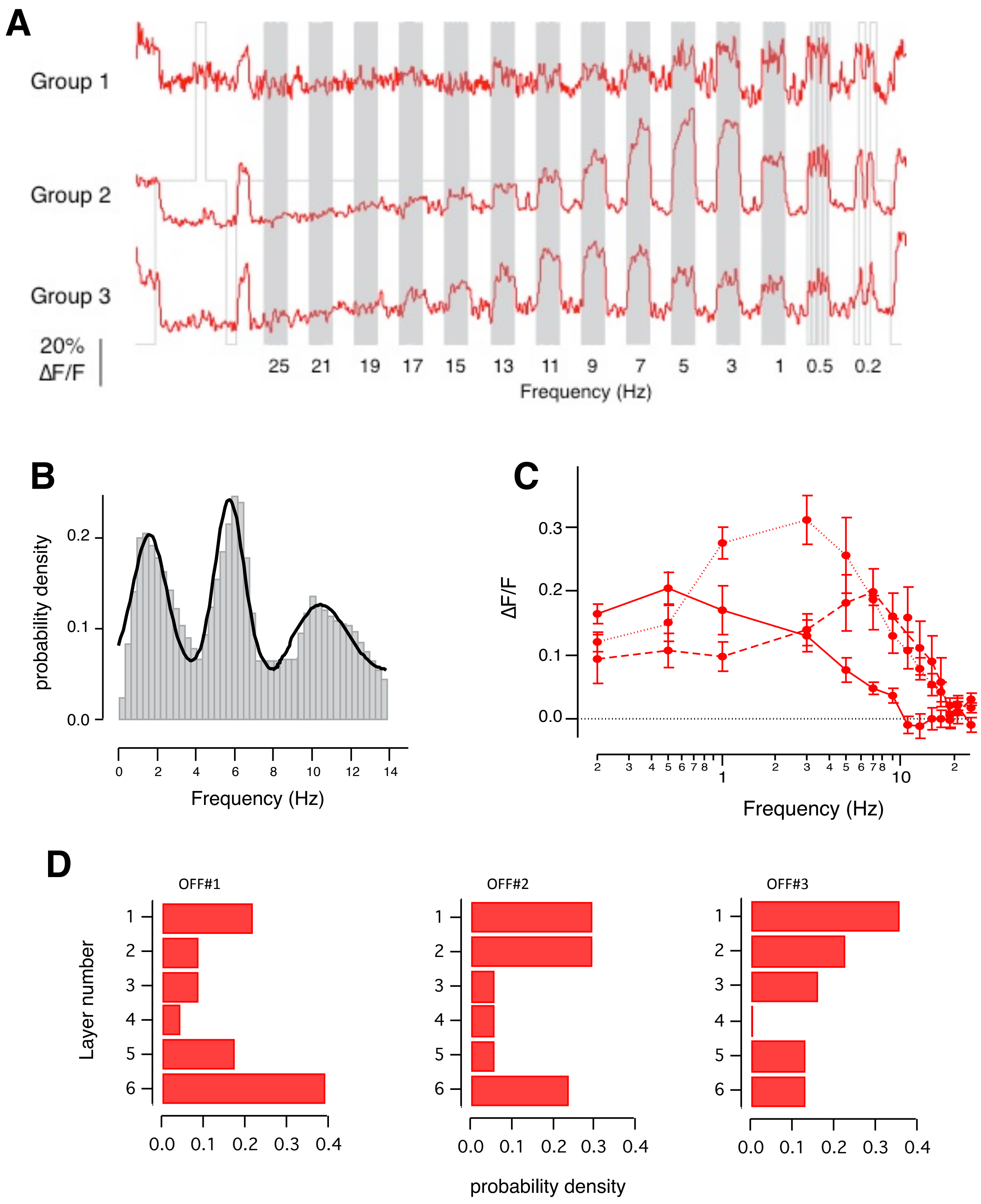
A
Group 1
Group 2
Group 3
20%
ΔF/F
25 21 19 17 15 13 11 9 7 5 3 1 0.5 0.2
Frequency (Hz)
B
probability density
0.0
0.1
0.2
0 2 4 6 8 10 12 14
Frequency (Hz)
C
ΔF/F
0.0
0.1
0.2
0.3
1
10
Frequency (Hz)
D
OFF#1
OFF#2
OFF#3
Layer number
1
2
3
4
5
6
0.0 0.1 0.2 0.3 0.4
probability density

Figure S3

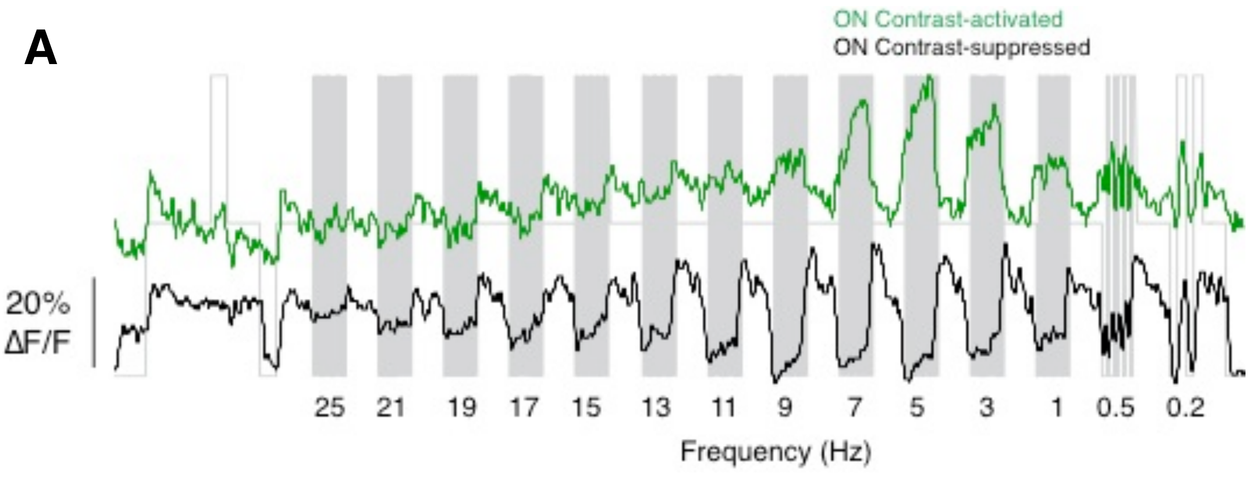
A
ON Contrast-activated
ON Contrast-suppressed
20%
ΔF/F
25
21
19
17
15
13
11
9
7
5
3
1
0.5
0.2
Frequency (Hz)

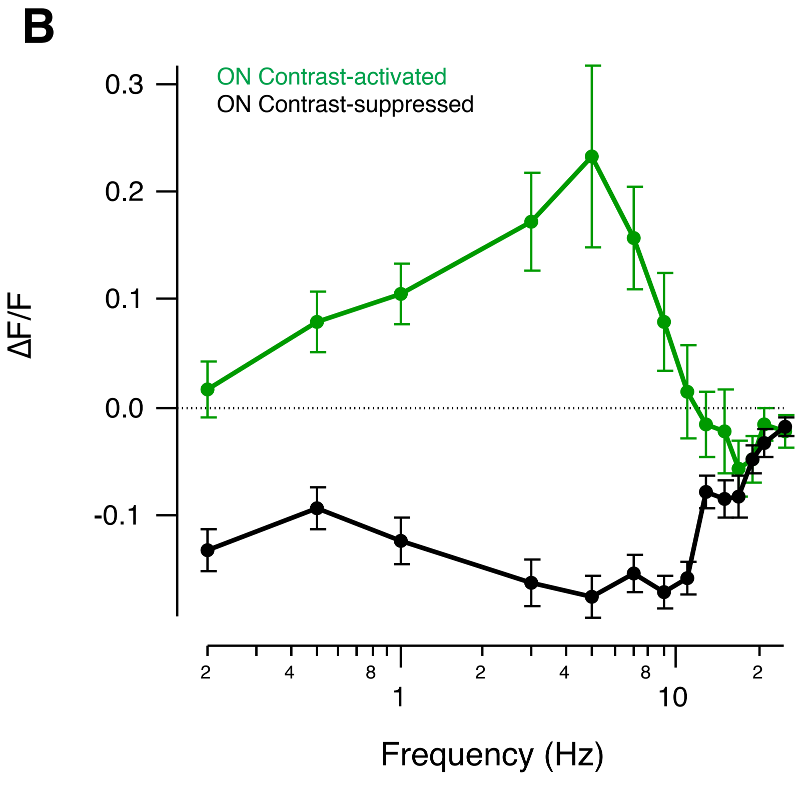
B
ON Contrast-activated
ON Contrast-suppressed
0.3
0.2
0.1
0.0
-0.1
ΔF/F
2
4
8
1
2
4
8
10
2
Frequency (Hz)

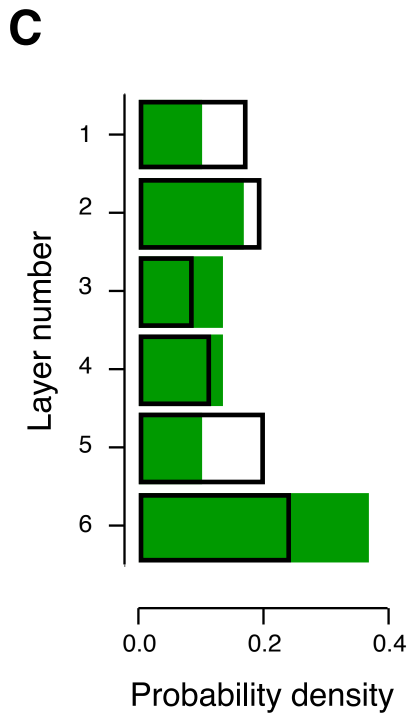
C
1
2
3
4
5
6
Layer number
0.0
0.2
0.4
Probability density

Figure S4

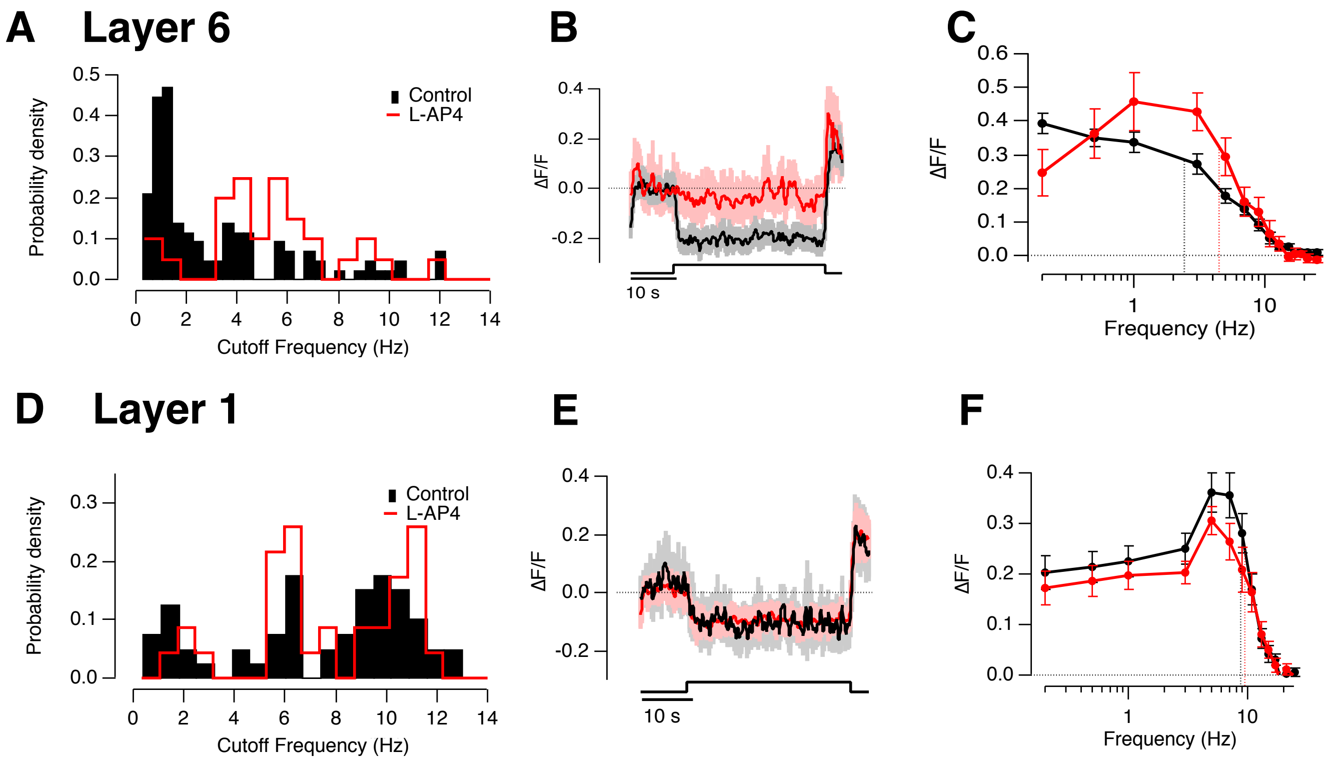
A
Layer 6
Probability density
Control
L-AP4
Cutoff Frequency (Hz)
B
ΔF/F
10 s
C
ΔF/F
Frequency (Hz)
D
Layer 1
Probability density
Control
L-AP4
Cutoff Frequency (Hz)
E
ΔF/F
10 s
F
ΔF/F
Frequency (Hz)

Figure S5

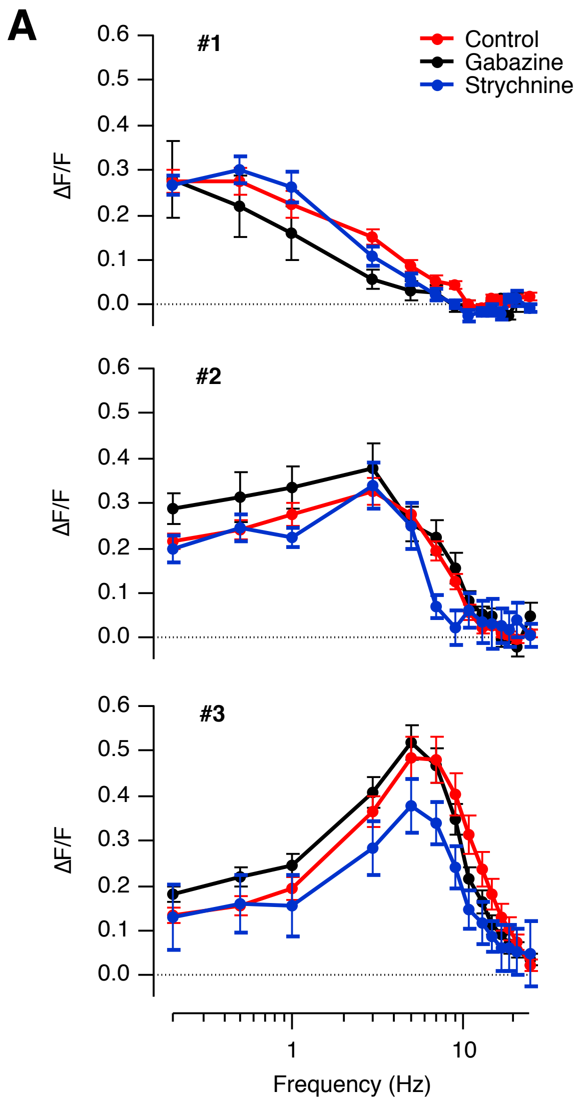

A
#1
#2
#3
Control
Gabazine
Strychnine
ΔF/F
0.6
0.5
0.4
0.3
0.2
0.1
0.0
1
10
Frequency (Hz)
